# Transformation Foci in IDH1-mutated Gliomas Show STAT3 Phosphorylation and Downregulate the Metabolic Enzyme ETNPPL, a Negative Regulator of Glioma Growth

**DOI:** 10.1038/s41598-020-62145-1

**Published:** 2020-03-26

**Authors:** N. Leventoux, M. Augustus, S. Azar, S. Riquier, J. P. Villemin, S. Guelfi, L. Falha, L. Bauchet, C. Gozé, W. Ritchie, T. Commes, H. Duffau, V. Rigau, J. P. Hugnot

**Affiliations:** 1 Institute for Neurosciences of Montpellier, Institut National de la Santé et de la Recherche Médicale (INSERM). U1051 - Hôpital Saint-Éloi, 80 Avenue Augustin Fliche - 34091 Montpellier cedex 5, Montpellier, France; 2 Keio University School of Medicine, Physiology Department. 35 Shinanomachi, Shinjuku-ku, Tokyo, 160-8582 Japan; 3 Institute for Regenerative Medicine & Biotherapy, Hôpital Saint-Éloi, 80 Avenue Augustin Fliche - 34091 Montpellier cedex 5, Montpellier, France; 4 Institute of Human Genetics, Centre National de la Recherche Scientifique (CNRS). UMR9002, 141 rue de la Cardonille, 34000 Montpellier, France; 5 Neurosurgery Department. Hôpital Gui de Chauliac, 80 Avenue Augustin Fliche, 34295 Montpellier, France; 6 Laboratory of Solid Tumors Biology. Hôpital Lapeyronie, 371 Avenue du Doyen Giraud, 34295 Montpellier, France; 7 Department of Pathology and Oncobiology. Hôpital Gui de Chauliac, 80 Avenue Augustin Fliche, 34295 Montpellier, France; 8 University of Montpellier, Place Eugène Bataillon, 34095 Montpellier, Cedex 05 France

**Keywords:** Cancer, CNS cancer

## Abstract

IDH1-mutated gliomas are slow-growing brain tumours which progress into high-grade gliomas. The early molecular events causing this progression are ill-defined. Previous studies revealed that 20% of these tumours already have transformation foci. These foci offer opportunities to better understand malignant progression. We used immunohistochemistry and high throughput RNA profiling to characterize foci cells. These have higher pSTAT3 staining revealing activation of JAK/STAT signaling. They downregulate RNAs involved in Wnt signaling (*DAAM2, SFRP2*), EGFR signaling (*MLC1*), cytoskeleton and cell-cell communication (*EZR, GJA1*). In addition, foci cells show reduced levels of RNA coding for Ethanolamine-Phosphate Phospho-Lyase (*ETNPPL/AGXT2L1*), a lipid metabolism enzyme. ETNPPL is involved in the catabolism of phosphoethanolamine implicated in membrane synthesis. We detected ETNPPL protein in glioma cells as well as in astrocytes in the human brain. Its nuclear localization suggests additional roles for this enzyme. *ETNPPL* expression is inversely correlated to glioma grade and we found no ETNPPL protein in glioblastomas. Overexpression of ETNPPL reduces the growth of glioma stem cells indicating that this enzyme opposes gliomagenesis. Collectively, these results suggest that a combined alteration in membrane lipid metabolism and STAT3 pathway promotes IDH1-mutated glioma malignant progression.

## Introduction

Diffuse low-grade gliomas (DLGG, WHO grade II) is a subtype of glial brain tumours typically affecting young patients in their third or fourth decades^1^. Compared to glioblastomas, much less is known about these tumours. They are mostly found in the brain white matter and are probably derived from transformation of oligodendrocyte progenitors which are the main proliferative cell population in the brain. They are most often found in functional brain areas such as the motor cortex, Broca’s area, frontal lobe and insula. Histologically, diffuse low-grade gliomas are divided into astrocytomas and oligodendrogliomas as tumoral cells look like normal brain astrocytes or oligodendrocytes^2^. However, recent works based on single-cell RNA-sequencing approaches have revealed that diffuse low-grade gliomas are in fact composed of at least two tumoral cell types with similarities to astrocytes and oligodendrocytes^3,4^. With regards to the mutational landscape, one recurrent DNA alteration found in 80% of patients is a missense mutation for the IDH1 gene (isocitrate dehydrogenase) involved in metabolism and epigenetic regulations^5^. Astrocytomas have an additional mutation for the ATRX gene whereas oligodendrogliomas show a 1p19q co-deletion. These mutations negatively influence the differentiation of progenitors which then accumulate in the brain. Treatment of these tumours consists of a maximal surgical resection often performed in awake-condition so as to preserve brain functional areas^6^. However, owing to the infiltrative nature of these tumours, it is not possible to remove all tumoral tissue and the tumour inexorably relapses. After an initial slow growth (2 mm per year), diffuse low-grade gliomas inescapably progress into rapidly-growing tumours, namely grade III gliomas or glioblastomas. The mutational landscape of these high-grade gliomas derived from low-grade lesions has been fully explored recently^7,8^. This malignant transformation then leads to rapid patient death. The duration of transition from low-grade to high-grade glioma is highly variable between patients. This can be very short (6 months) or very long (>15 years). Currently, there is no good predictive tool to evaluate this time and this poses important problems for clinicians to monitor the patients and give appropriate treatments. If patients could be identified with increased risk for progression, they might be offered early adjuvant treatment or targeted new therapeutic approaches. In order to do so, we need to understand in depth, the early molecular mechanisms by which an initial diffuse low-grade glioma progresses into a higher grade, a process which is still unclear. One major difficulty to characterize this initial malignant progression is to be able to capture the key transformation steps occurring in the tumour. We attempted to fill this gap by extensive examination of diffuse low-grade glioma sections. This led us to identify foci of hypercellularity in diffuse low-grade gliomas^9^. These foci are present in 20% of patients. Importantly, these patients have a worse prognosis compared to foci-free patients. We reported a first partial characterization of the cellular and DNA alterations found in these foci using IHC and FISH. We found that foci cells 1- have a higher mitotic index (Ki67^+^ cells) and vascular density, 2- can show sign of hypoxia (HIFα staining), 3- can have frequent chromosome 7 polysomy as well as higher intensity for EGFR and AKT stainings compared to the rest of the tumour. These alterations strongly suggest that these foci are composed of cells undergoing further transformation events.

In this article, we wanted to understand further how diffuse low-grade gliomas progress into high-grade gliomas by characterizing early histological signs of transformation. We thus focused our study on these foci which are likely candidates of early transformation. Our objectives were 1/to study pathways which might allow formation of these foci, 2/to identify genes whose expression is dysregulated in these foci, 3/to perform functional analysis of one identified gene *in vitro*. We used IHC and high throughput RNA profiling to characterize these foci in 8 tumours. This led us to identify a metabolic enzyme (ETNPPL, Ethanolamine-Phosphate Phospho-Lyase) which was further studied at the functional level.

## Material and Methods

### Selection of patients

All methods and all experimental protocols used in this article were carried out and approved in accordance with relevant guidelines and regulations of the French “Institut National de la Santé et de la Recherche Médicale” (INSERM). Glioma samples were obtained from the “Centre de Ressources Biologiques” (Collection NEUROLOGIE (8) DC-2013-2027/DC-2010-1185/Authorization AC-2017-3055/Research Protocol P487) and our research approved by the Institutional Review Board (2019_IRB-MTP_10-15) of the Montpellier Hospital. Informed and written consents were obtained from the patients before using their samples. Gliomas were graded by a neuropathologist (Pr V. Rigau) and cases with foci were selected according to criteria reported in^9^ e.g. (1) higher cellular density compared to the rest of the tumour, (2) nuclear atypia with or without anisocaryosis, (3) enhanced vascularization of the foci with early signs of endothelial proliferation. Clinical and tumour pathological features are summarized in Table S1. For RNA extraction, samples were obtained from patients who had a first surgery and no pre-operative therapies. IDH1 mutation was established by immunohistochemistry against the IDH1 R132H epitope with at least 80% of positive cells in both DLGG and foci for each tumour. IDH1 mutation was then confirmed by sequencing of the IDH1 gene exon 4. 1p/19q mutation was identified by molecular detection of loss of heterozygosity using polymorphic markers to screen both whole 1p and 19q chromosome arms as described in^10^. ATRX mutation was established by loss of nuclear staining with IHC using ATRX polyclonal antibody (SIGMA Ref HPA001906). To explore the expression of ETNPPL by immunofluorescence in the non-tumoral human, we used a cortical sample removed during neurosurgical approach for meningioma resection.

### Immunohistochemistry (IHC) and Immunofluorescence (IF)

All antibody references and dilutions are indicated in Table S2. IHC for pSTAT3 and ETNPPL were performed on paraffin-embedded tumours fixed with formalin 37% (FFPE) for 1 day. Stainings were done using 4 μm sections and the Leica Biosystems’ Bond Polymer Refine Detection kit (DS9800) after suitable epitope retrieval with citrate buffer and according to manufacturer’s protocol. Immunofluorescences were done on frozen sections of tumours fixed with paraformaldehyde 4% for 1 hour and cryopreserved in successive sucrose solutions (10%, 20%, 30%, 24 h each). Sections (10 μm) were permeabilized and saturated with 3% triton X-100 and 10% donkey serum diluted in PBS 1X and then incubated overnight with primary antibodies. Secondary conjugated antibodies Alexa 488 or Cy3 (Jackson & Molecular Probe) were incubated for 90 minutes. Incubations without primary antibody or with antibodies against GFP were used as negative controls to avoid misinterpretation due to the presence of autofluorescent compounds (lipofuscin). To check ETNPPL antibody specificity, the blocking peptide (Novus NBP1-91655PEP) was incubated with the antibody (ratio of 10:1 peptide/antibody) at room temperature for one hour before the staining.

### Equipment and settings

Bright field images were taken with a Nikon Eclipse microscope and a NIH-elements software using standard settings. Fluorescent images were taken with a Zeiss apotome Axio Imager 2 equipped with a Zeiss ZEN software. Main settings were: binning 2 × 2, apoptome mode: 5. Quantifications were obtained by two investigators (N.L., M.A.) on image captures and with ImageJ software manual cell counting tool. For western blots, proteins were detected using an Odyssey CLx Li-Cor apparatus and accompanying software. Exposure times were 7 minutes.

### RNA profiling

Tumours with foci of at least four millimeters in diameter, assessed by hematoxylin & eosin stainings and with at least 80% of IDH1 R132H positive cells were selected. Four drills (two in foci and two in the other part of the tumour – Fig. S2B) were performed in the FFPE tumour blocks using a two millimetres punch from a Tissue Micro Array apparatus (Master 3DHistech LTD, Hungary) in RNAse-free conditions. After the punches, the adequate selection of tumour areas was checked by hematoxylin & eosin stainings of sections (Fig. S2B). Total RNA was extracted using the Qiagen RNeasy FFPE kit, quantified with Nanodrop 1000 (Thermo Fisher) and the RNA integrity number (RIN) was determined using a Bioanalyzer 2100 (Agilent Technologies). The RIN was on average 2.5 thus indicating degraded RNA. This was expected using FFPE samples but the RNA were still suitable for labelling and hybridization on DNA chips according to the Affymetrix technical department. After amplification and labelling with an Affymetrix WT Pico Kit, cDNA were hybridized on Human Gene 2.1 ST chips. Arrays CEL files were processed with Affymetrix Expression Console software (GC-RMA (Robust Multiarray Average) algorithm) and Affymetrix Transcriptome Analysis Console (TAC 3.1.0.5) softwares. Array CEL files were also analyzed in parallel using R software 3.6. In that case, files were preprocessed by RMA algorithm from the Oligo R Package version 1.48.0. Then Limma package version 3.40.6 was used to identify genes differentially expressed. Genes identified both by Transcriptome Analysis Console 3.1.0.5 and Limma algorithms and with a p-value ≤ 0.05 (unpaired tests) and a fold change ≥1.3 (DLGG/foci) were considered as differentially expressed (Table 1). The raw data that support the findings of our study are openly available at the functional genomics data Gene Expression Omnibus (GEO: GSE130149).

**Table 1:** Identification of dysregulated genes in foci.

Gene Symbol	Description	DLGG (log2)	Foci (log2)	Fold Change (DLGG/foci)	p-value
***SFRP2***	Secreted Frizzled Related Protein 2	6.51	5.5	2	0.0475
***CST3***	Cystatin C	8.94	8.1	1.79	0.0277
***DAAM2***	Dishevelled Associated Activator Of Morphogenesis 2	7.2	6.4	1.75	0.0253
***ETNPPL***	Ethanolamine-Phosphate Phospho-Lyase	6.01	5.22	1.72	0.0096
***TMEM47***	Transmembrane Protein 47	6.16	5.48	1.61	0.0126
***MLC1***	Megalencephalic Leukoencephalopathy With Subcortical Cysts 1	6.86	6.23	1.54	0.0028
***KCNN3***	Potassium Calcium-Activated Channel Subfamily N Member 3	6.42	5.8	1.54	0.0377
***ADCYAP1R1***	Adenylate Cyclase Activating Polypeptide 1 (Pituitary) Receptor Type I	7.28	6.72	1.47	0.0116
***GJA1***	Gap Junction Protein Alpha 1	5.79	5.27	1.43	0.0223
***EZR***	Ezrin	6.98	6.46	1.43	0.0048
***ALDOC***	Aldolase, Fructose-Bisphosphate C	7.81	7.36	1.37	0.0107
***ATP1A2***	ATPase Na + /K + Transporting Subunit Alpha 2	6.88	6.47	1.33	0.0072
***SLC1A3***	Solute Carrier Family 1 Member 3	5.96	5.57	1.32	0.0273

Fold changes (DLGG/foci) are indicated. p-values (ANOVA unpaired tests, n = 8 cases) are indicated.

### RT-qPCR

cDNA synthesis was performed using 1–5 μg of total RNA with random hexamers and reverse transcriptase (Promega, GoScript). Quantitative RT-qPCR was performed in triplicate for each tumour sample using the KAPA SYBR PCR kit (Sigma Aldrich KK4600) with a LightCycler 480 apparatus (Roche). 1 μL of cDNA was used for each reaction. Primers are listed in Table S2. Expression values were calculated using the 2^−ΔΔCT^ method and normalized using the housekeeping gene RPLP0.

### Comparative genomic hybridization array (CGHa)

The LGG85 cell line was established from a male patient diagnosed with a grade-IV IDH1-mutated glioma with culture conditions used for Gli4 and Gli7 glioblastoma cell lines^11^. Array CGH profiling for the LGG85 line and from the original tumour were performed with the Human Agilent Sureprint G3 8 60 K Microarray Kit (Agilent Technologies, USA). LGG85 DNA was labelled with cyanin 5 (Cy5) while reference DNA from a male subject was labelled with cyanin 3 (Cy3). Sample and reference DNAs were pooled and hybridized for 24 hours at 67 °C on the arrays. The fluorescence was read by the Agilent SureScan Microarray scanner and the Cy5 /Cy3 ratios were converted into log2 transformed values with the Agilent Cytogenomics software. DLRS (Derivative Log Ratio Spread), gRepro, rSignalIntensity and LogRatioImbalance were 0.2, 0.009, 450.68 and −0.1 respectively. A single copy loss was associated with a value of the log2 below −0,8 (log2 (1/2) = −1) since a single copy gain was associated with a value of the log2 above 0,5 (log2 3/2) = 0,58).

### Lentiviral vectors and glioma cell culture

To study the effect of ETNPPL overexpression, we designed two lentiviral vectors combining an EGFP-T2A-Puromycine resistance gene and the coding sequence for Human ETNPPL or Luciferase, controlled by a doxycycline-regulated Tre3G promoter (Vectorbuilder). A third lentivirus was built to express a blasticidin resistance gene and the doxycycline-regulated Tet-on activator. Mycoplasma-free Gli4, Gli7 and LGG85 cells were cultured as described^12^ on poly-HEMA coated vessels and in defined media (DMEM/F12 1:1 (Invitrogen) supplemented with N2 (Invitrogen), 2 mM glutamine (Invitrogen), 2 μg/mL heparin (Sigma), 10 ng/mL EGF (Peprotech) and 10 ng/mL FGF2 (Peprotech)). 100,000 dissociated cells were infected with the Luciferase-EGFP or ETNPPL-EGFP viruses (MOI 7), grew for two weeks and transduced cells were selected by sorting for green fluorescence (GFP) (Aria cytometer BD). Cells were then infected with the Tet3G lentivirus and selected for 2 weeks with blasticidin (0.5 μg/mL). ETNPPL expression was induced by doxycycline 1 μg/mL and checked with ETNPPL antibody by WB and by immunofluorescence on cells seeded on poly-D-lysine/laminin coated coverslips. Effect of ETNPPL on cell growth was measured by seeding 5,000 infected cells per well in 1 mL of media in 24-well plates. After 8 days, the number of cells were determined by dissociation with trypsin and automatic counting with Z2 counter (Beckman Coulter). For EdU incorporation, cells were incubated with EdU 10 μM for 4 hours and processed for staining following manufacturer’s recommendation (Baseclick kit). Explant cultures of IDH1-mutated diffuse low-grade gliomas used to explore the expression of ETNPPL are described in^12^.

### Western blots

Total proteins from tissue samples and cultured cells were obtained and used for Western Blot as described by^12^. Proteins were detected using the Odyssey CLx Li-Cor technology. Briefly, primary antibodies were incubated in Li-Cor PBS buffer overnight at 4 °C. After washing, membranes were incubated with secondary fluorescent dye (IRDye 800CW for ETNPPL and IRDye 680LT for β-actin).

### Statistical analysis and countings

All statistical tests used and sample sizes (n) are indicated in the figure legends. Tests were performed using the GraphPad Prism software (v8.2.1). Significances: ****, ***, **, * represent p < 0.0001, p < 0.001, p < 0.01 and p ≤ 0.05 respectively. For cell countings, at least 5 independent fields of 10,000 μm² were counted. The effect of ETNPPL overexpression in Gli4, Gli7 and LGG85 cell was performed in 3 independent experiments.

## Results

### Foci show activated STAT3 pathway and dysregulated gene expression

**Figure 1:**
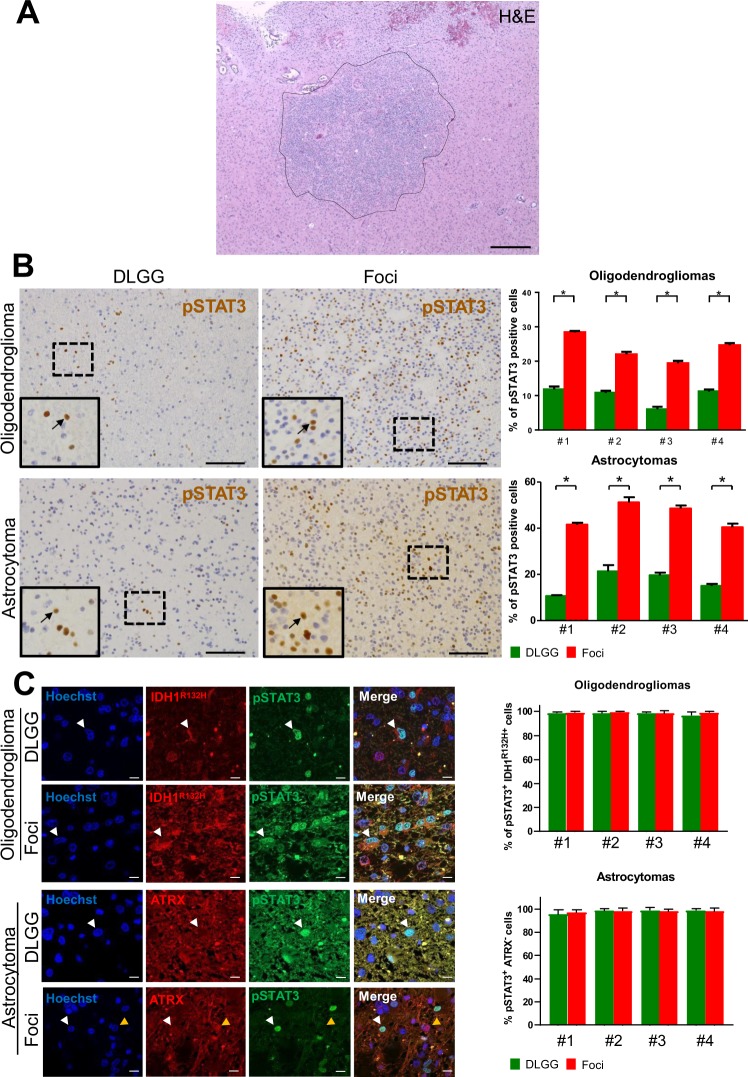
pSTAT3 stainings in diffuse low-grade glioma foci. (**A**) Example of a high cell density foci detected in a diffuse low-grade glioma. H&E: hematoxylin and eosin stainings. Scale bar 500 μm. **(B)** Representative stainings for pSTAT3 in diffuse low-grade gliomas. Left and right-hand images are taken outside and inside the foci, respectively. Images taken in an oligodendroglioma (upper images) and an astrocytoma (lower images) are presented. Arrows point to pSTAT3^+^ cells in each inset. Scale bars 150 μm. Quantification of pSTAT3^+^ cells detected in 5 fields (10,000 μm²) outside and inside the foci in 4 oligodendrogliomas and 4 astrocytomas are presented on the right-hand side. Test = Mann-Whitney tests n = 5, p value = 0.011 for oligodendrogliomas and astrocytomas. **(C)** Expression of pSTAT3 by tumoral cells. Representative photographs of double immunofluorescences for IDH1^R132H^/pSTAT3 and ATRX/pSTAT3 in one grade II oligodendroglioma and one grade II astrocytoma, in and outside the foci. White arrows point to IDH1^R132H+^/pSTAT3^+^ (oligodendroglioma) and ATRX negative/pSTAT3^+^ (astrocytoma) cells. The yellow arrow on astrocytoma photograph indicates one example of an ATRX^+^ cell to show the quality of the immunofluorescence. Right-hand histograms show the percentage of tumoral pSTAT3^+^ cells (e.g. pSTAT3^+^ cells which are positive for IDH1 R132H or negative for ATRX) outside and inside the foci assessed by IDH1^R132H^/pSTAT3 and ATRX/pSTAT3 co-stainings in 4 oligodendrogliomas and 4 astrocytomas (5 fields (10,000 μm²) outside and inside the foci quantified per patient). Only cells with obvious nuclear pSTAT3 staining were counted.

To molecularly characterize foci found in DLGG patients (Fig. 1A), we selected 8 tumours with mutations for IDH1 (IDH1 R132H) with and without 1p19q co-deletion (4 oligodendrogliomas and 4 astrocytomas respectively) (Table S1). These 8 cases contained foci which were selected on the basis of a local increase in cell density (Figs. 1A, S1). During tumorigenesis, canonical pathways controlling cell proliferation and fate are frequently dysregulated. We explored this possibility by using IHC for Notch, STAT3, BMP, and Ras signalings. We assessed the expression and subcellular location of key proteins for these pathways, respectively activated Notch (NICD), pSTAT3 (phosphorylated on tyrosine 705), BMP4 and p-ERK. Only pSTAT3 stainings showed a clear modification in the foci compared to the rest of the tumour (Fig. 1B). The eight studied tumours showed a small number of scattered pSTAT3^+^ cells, however a sharp and statistically-significant increase of their percentage was observed in foci (Fig. 1B). To ascertain that these cells were tumoral and not cells of the tumour environment, we performed double labelling for pSTAT3 and proteins which are frequently altered in DLGG, namely the mutated form of IDH1 (R132H) and loss of ATRX. In the 4 examined oligodendrogliomas (Fig. 1C), we found that >90% of pSTAT3^+^ cells in the foci or in the rest of the tumour also expressed the mutated form of IDH1 R132H. Expression of IDH1 R132H assessed by IF in the astrocytomas was too weak to perform reliable double pSTAT3/IDH1 R132H stainings so, as an alternative, pSTAT3/ATRX stainings were done. Figure 1C shows that in the 4 astrocytomas, most of pSTAT3^+^ cells in or outside the foci have lost ATRX expression (>90% of cells). Taken together, these results provide evidence that tumoral cells in foci increase STAT3 signaling.

We then investigated whether we could identify genes whose expression is specifically dysregulated in foci compared to the rest of the tumour. We used FFPE blocks to microdissect a tumoral region within or outside the foci for the 8 tumours (Fig. S2B). RNAs were extracted and profilings were performed. The RNA quality obtained from hospital FFPE blocks precluded the use of a RNA sequencing approach so DNA microarrays were used instead. Clustering analysis performed with the 5,000 top-expressed genes indicates that the tumour and its foci (with the exception of tumour 3) are closely transcriptionally-related and cluster together (Fig. S3).

To identify differentially-expressed genes, the statistical analysis was performed using the 8 tumours together to increase statistical power. A fold change value of 1.3 was selected as a more selective threshold retrieved very few genes. Table 1 shows a list of the 13 identified genes. All were downregulated in foci. Literature analysis shows that these genes are implicated in various pathways and cellular processes such as Wnt signaling (*DAAM2, SFRP2*), glutamate transport (*SLC1A3*), metabolism (*ALDOC, ETNPPL*), cytoskeleton (*EZR*), cell-cell adhesion (*GJA1*) and cyclic AMP pathway (*ADCYAP1R1*).

**Figure 2:**
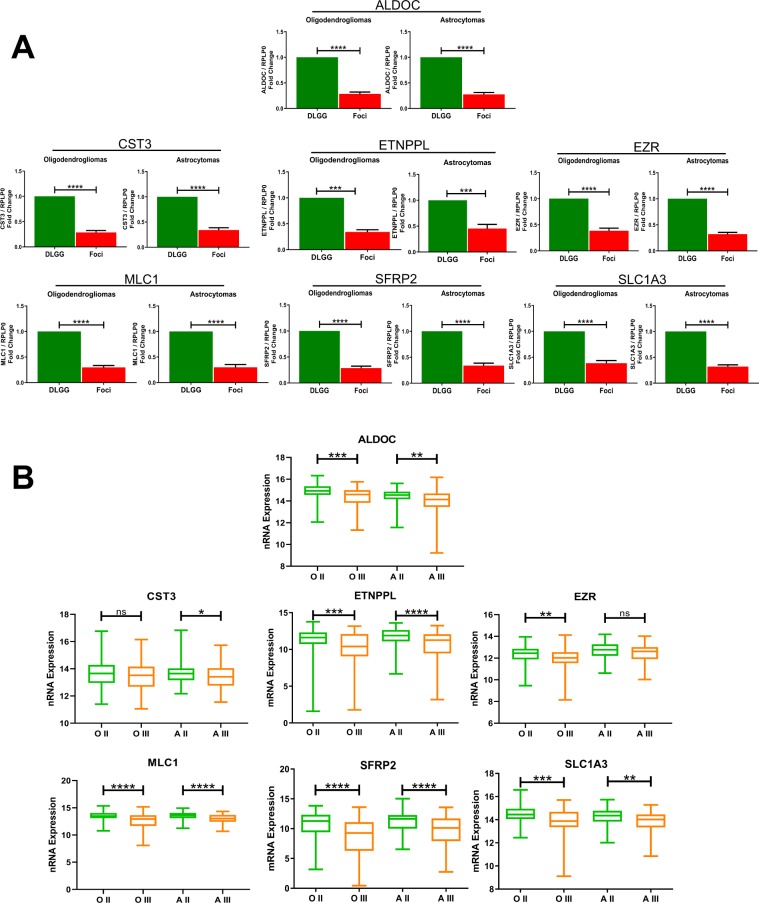
qPCR validation of differentially-expressed genes and expression in grade II and III gliomas. (**A**) qPCR quantification of indicated RNAs in foci and in the other part of the tumour (DLGG) in oligodendrogliomas and astrocytomas. Values represent fold change (foci area/non foci area) of indicated RNA expression normalized by RPLP0 quantification. n = 10 tumours. Test = Wilcoxon matched-pairs test. **(B)** Expression of indicated genes in grade II and grade III gliomas retrieved from the TCGA database. A similar analysis done for all identified genes is presented in Table S3. OII (Oligodendroglioma grade II, 108 patients). OIII (Oligodendroglioma grade III, 76 patients). AII (Astrocytoma grade II, 87 patients). AIII (Astrocytoma grade III, 98 patients). Tests = Kruskal-Wallis with a Dunn post-hoc test.

In order to validate these results with an independent approach, we performed RT-qPCR on 10 tumours (those used for RNA profiling + 2 additional tumours). We selected 7 genes which are involved in EGFR signalling (*MLC1*), metabolism (*ALDOC, ETNPPL*), Wnt signaling (*SFRP2*), protease inhibition (*CST3*), skeleton function (*EZR*) and glutamate transport (*SLC1A3*). Figure 2A shows a significant downregulation of these 7 genes in the foci of the 10 analysed tumours. These results corroborate those obtained with the DNA microarray technology.

There was a possibility that the genes identified as differentially-expressed were in fact the consequence of a different number of non tumoral cells (for instance immune or stromal cells) in foci areas versus the rest of the tumour. We explored this hypothesis using two approaches. First we measured the percentage of tumoral cells in or outside the foci with IHC against IDH1 R132H (Fig. S2A). We found that both areas were mainly composed of tumoral cells (>80%) and no statistically-significant difference can be detected (Fig. S2C). Second, we used a bioinformatics approach using gene expression signatures to infer the fraction of stromal and immune cells in tumour samples as described in^13^. This method called ESTIMATE (Estimation of STromal and Immune cells in MAlignant Tumours using Expression data) calculates scores (between 0 and 1) predictive of tumour infiltration by stromal and immune cells and consequently, of tumour purity. All samples (8 tumours + 8 foci) were analyzed by ESTIMATE and results presented in Fig. S2D show that tumour and foci purity were very high (>0.8). No statistical differences in purity could be detected between the tumour and the foci areas (Fig. S2D). These analyses indicate that the genes identified as differentially-expressed genes are very likely expressed by tumoral cells.

We then explored whether the genes dysregulated in foci were also differentially-expressed with tumoral progression. To this aim, we retrieved the expression value for the 13 identified genes from the cancer genome atlas (TCGA)^14^ in grade II and grade III gliomas. Statistical analyses performed in IDH1-mutated tumours show that these genes were differentially-expressed between both type of grade II and III gliomas (Table S3). Examples of TCGA gene expression for the 7 quantitative PCR-validated genes (Fig. 2A) are presented on Fig. 2B.

These results suggest that the observed dysregulation of genes in foci account for a malignant progression taking place in this part of the tumour.

### Glioma cells express the ETNPPL lipid metabolism enzyme

**Figure 3:**
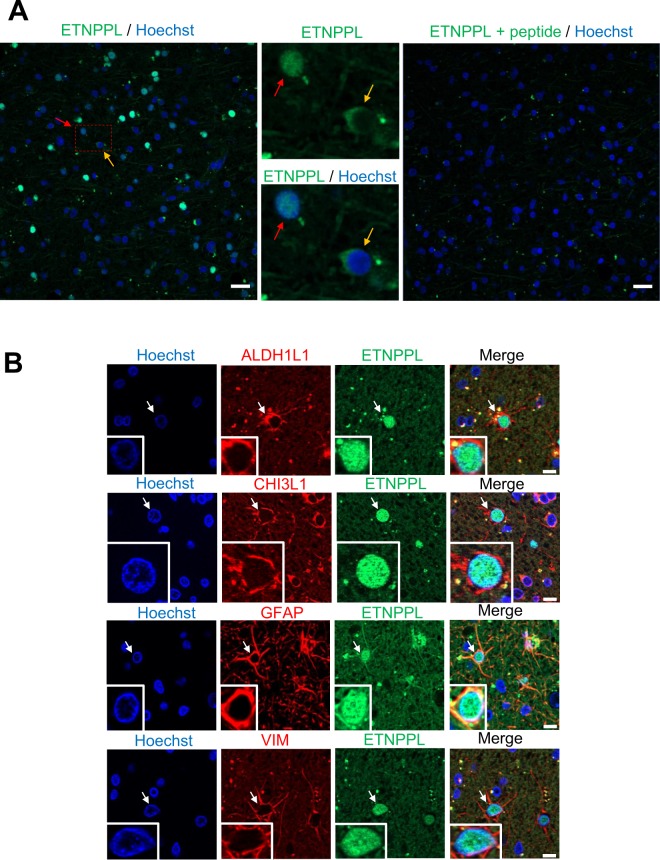
Detection of ETNPPL protein in human cortex. (**A**) Immunofluorescence for ETNPPL (green) in human cortex sections. The two middle photographs are high magnification of red dotted-line square on the left-hand image. ETNPPL is detected in the cytoplasm (yellow arrow) or the nucleus (red arrow). The right-hand image shows the absence of detection of ETNPPL when the antibody was pre-incubated with the ETNPPL peptide used to generate the antibody. Remaining green dots are likely to be autofluorescent lipofuscin which is often present in human brain samples. Scale bars = 20 μm. **(B)** Immunofluorescences for ETNPPL (green) and indicated proteins (red) in human cortex. ETNPPL is detected in the nucleus of cells expressing ALDH1L1, CHI3L1, GFAP and VIM (vimentin), identifying these cells as astrocytes. Arrows point to double positive cells. Scale bars = 10 μm.

**Figure 4:**
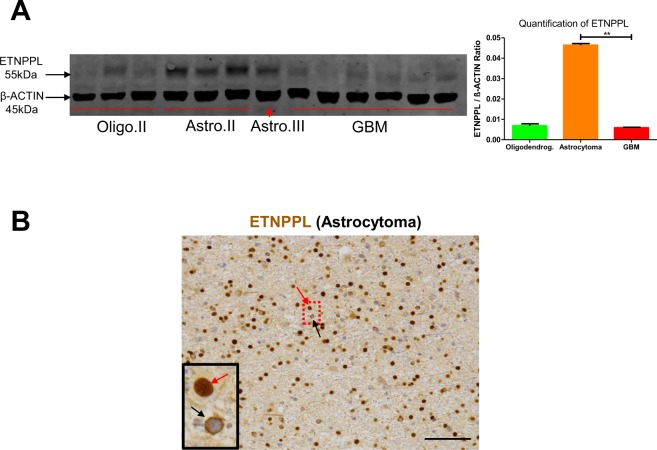
Detection of ETNPPL in gliomas. (**A**) WB detection of ETNPPL in proteins extracted from indicated gliomas. ETNPPL is detected as a single band with the predicted size (55 kDa). The uncropped image of the WB is presented on Supplemental Fig. 8A. Oligo II: grade II oligodendrogliomas, Astro II and III: grade II and III astrocytomas. GBM: IDH1 non-mutated glioblastomas. β-actin detection was used for normalization. Quantification for ETNPPL is presented on the right-hand side panel (4 astrocytomas IDH1 R132H-mutated and 6 glioblastomas). Test = Mann-Whitney test (**p = 0.01). **(B)** Immunohistochemistry for ETNPPL (brown) in one IDH1-mutated diffuse grade II astrocytoma. The protein is present either in the nucleus (red arrow) or in the cytoplasm (black arrow) of cells. Scale bars = 150 μm. Nuclei are stained with hematoxylin.

**Figure 5:**
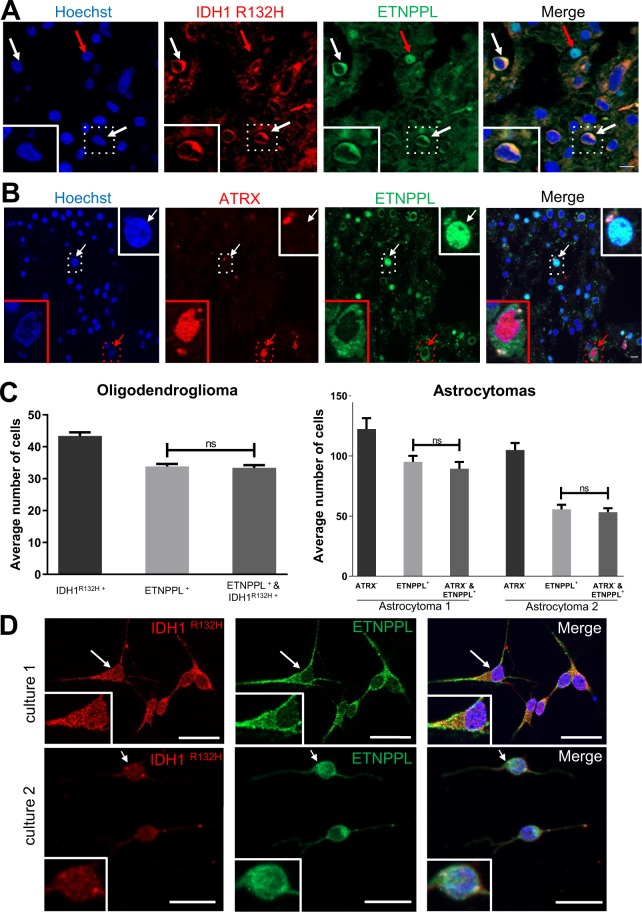
Expression of ETNPPL by tumoral cells. (**A**) Representative photograph of double immunofluorescence for IDH1^R132H^/ETNPPL in one grade II oligodendroglioma. White arrows point to IDH1^R132H^ positive tumour cells co-expressing ETNPPL in the cytoplasm (green). Red arrow points a nuclear ETNPPL expression in a non tumoral cell (IDH1^R132H^ negative) indicative of staining specificity. Scale bars 10 μm. **(B)** Representative photograph of double immunofluorescence for ATRX/ETNPPL in one grade II astrocytoma. Most of ETNPPL^+^ cells do not express ATRX and are thus tumoral. The red arrow points to an ETNPPL^+^ ATRX^+^ cell which is unlikely to be tumoral whereas the white arrow shows an ETNPPL^+^ ATRX^-^ tumoral cell. Insets are high magnification of pointed cells. Scale bars 10 μm. **(C)** Quantification of tumoral ETNPPL^+^ cells. Histograms show the average number of indicated cells by counted field (x40) in one grade II oligodendrogliomas and two grade II astrocytomas. In oligodendroglioma, ETNPPL^+^ cells are also IDH1 R132H^+^ whereas in the two astrocytomas they are ATRX^-^. Tests = Mann-Whitney tests are non-significant (n.s.) p = 0.74; 0.55; 0.53 for the 3 tumours. **(D)** Detection of ETNPPL (green) in 2 diffuse low-grade gliomas cultures. The presence of tumoral cells is indicated by staining for the mutated IDH1 R132H protein (red). Insets are high magnification of cells pointed with arrows. Note that in those two patients, the ETNPPL protein was mainly detected in the cytoplasm. Scale bars = 20 μm.

Lipid metabolism is altered in cancer cells^15^. The expression of a lipid metabolic enzyme called Ethanolamine-Phosphate Phospho-Lyase (ETNPPL, also known as AGXT2L1) was found reduced in foci (Table 1). ETNPPL is involved in the catabolism of phosphoethanolamine (Fig. S4A), a key compound for the synthesis of phosphatidylethanolamine which is a major phospholipid of the cellular membrane^16^. ETNPPL is mainly expressed in brain and in liver according to NCBI gene database (Fig. S4B)^17^. In human brain, its expression appears to be restricted to mature astrocytes (Fig. S4C)^18^. Little is known about ETNPPL (13 publications) and a potential link with brain tumours has not been established. This prompted us to explore the expression and role of ETNPPL in the human brain and gliomas. We started by performing IF for ETNPPL using one human cortical sample removed during neurosurgical approach for meningioma resection. The ETNPPL protein was detected by a fraction of cells either in the nuclei or in the cytoplasm (Fig. 3A, left-hand and central images). Immunofluorescence specificity was assessed by loss of signal if the antibody is pre-incubated with an ETNPPL peptide (Fig. 3A, right-hand image). To confirm the preferential expression of ETNPPL in astrocytes, double immunofluorescences were performed for four astrocytic markers (ALDH1L1, CHI3L1, GFAP, VIM). A very large fraction of ETNPPL^+^ cells (>95%, >450 cells counted for each markers) were positive for these four markers (Fig. 3B). Surprisingly for a metabolic enzyme, ETNPPL staining was located in the cell nuclei. ETNPPL antibody quality was indicated by the presence of a band at the expected size (55 kDa) on a western blot (WB) performed with DLGG extracts (Fig. 4A). These results confirmed the astrocytic expression of ETNPPL as suggested by RNA expression databases. We then analyzed ETNPPL expression in diffuse low-grade gliomas samples. By WB, ETNPPL was detected as a single band in 5 of the 6 analyzed grade II tumours (Figs. 4A and S8A). By IHC and reminiscent of what was observed in the normal brain, ETNPPL was detected either in the cytoplasm or the nuclei of the cells in one grade II astrocytoma (Fig. 4B). DLGG can contain normal and mutated cells so it was important to establish that ETNPPL^+^ cells were tumoral. This was addressed by performing double IF for ETNPPL and IDH1 R132H (1 oligodendroglioma) and ATRX (2 astrocytomas). As mentioned previously, expression of IDH1 R132H assessed by IF in the astrocytomas was too weak to be quantified accurately. In the oligodendroglioma (Figs. 5A,C, S6A,B), we found that >95% of ETNPPL^+^ cells also expressed the mutated form of IDH1 R132H. Figure 5B-C shows that in the 2 astrocytomas, most of ETNPPL^+^ cells have lost ATRX expression (>95% of cells). We also examined the ETNPPL expression in gliomas cells using DLGG primary cultures (Fig. 5D). In the two explored cultures, most of IDH1 R132H^+^ cells (>90%, 150 cells counted) co-expressed ETNPPL. In these 2 patients, the protein was mainly in the cytoplasm.

The ETNPPL expression in diffuse low-grade glioma cells was also validated using a second antibody (HPA072938) directed against another part of the protein (Table S2). The quality of this antibody was checked by WB and immunofluorescence with ETNPPL-overexpressing cells (see below) (Fig. S7A,B). Either cytoplasmic or nuclear expression of ETNPPL was detected in tumoral cells identified by the expression of IDH1R132H in one oligodendroglioma (Fig. S7C). ETNPPL was also detected with this antibody in the cytoplasm or the nucleus of diffuse low-grade gliomas cells in culture (Fig. S7D).

All together, these results demonstrate that ETNPPL is expressed by tumoral cells in DLGG.

### Foci and high-grade gliomas have a reduced expression of ETNPPL

**Figure 6:**
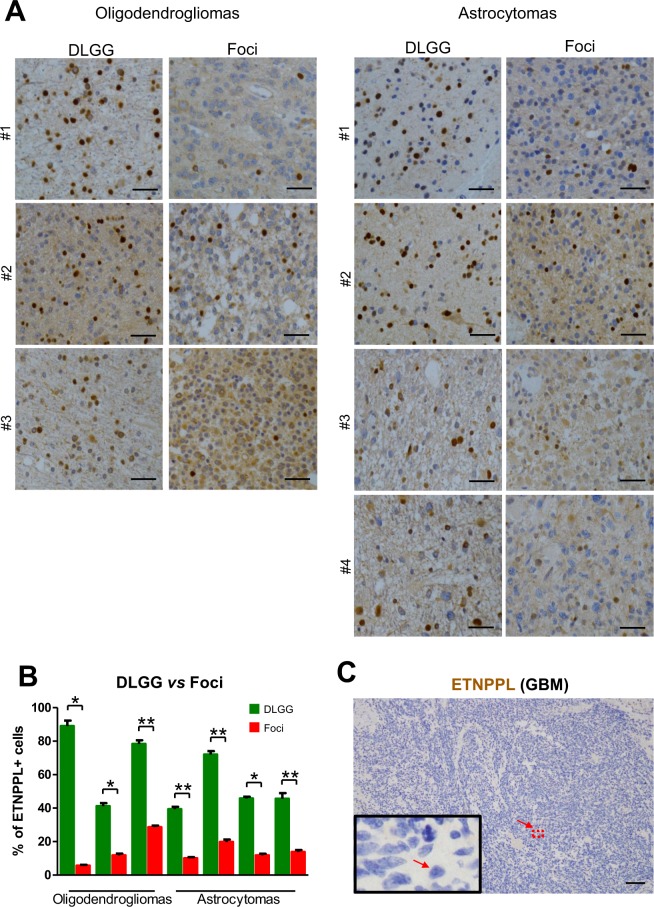
ETNPPL protein reduction in foci. (**A**) Representative photographs of ETNPPL immunohistochemistry (brown stainings with DAB) in 3 grade II oligodendrogliomas and 4 grade II astrocytomas. Photographs were taken in the foci and the rest of the tumour (DLGG). Nuclei are stained with hematoxylin. Scale bars = 50 μm. **(B)** Quantification of ETNPPL^+^ cells in the 3 oligodendrogliomas and 4 astrocytomas. The histograms represent the % of ETNPPL^+^ cells detected among all cells present per 10,000 μm² for each tumour (n = 5 fields). Tests = Mann-Whitney tests. **(C)** Immunohistochemistry for ETNPPL (brown) in one glioblastoma. The protein is not detected. Scale bars = 150 μm. Nuclei are stained with hematoxylin.

Microarray and qPCR analyses indicate that ETNPPL RNA is reduced in the foci (Table 1, Fig. 2A). First, we validated this at the protein level using IHC in seven DLGG containing foci. Figure 6A,B shows a significant reduction of the percentage of ETNPPL^+^ cells in the foci even if its expression is highly variable between tumours. Second, we also explored glioma databases for the expression of ETNPPL in different tumour grades. All three databases (TCGA, Rembrandt and NCBI dataset GDS1962) showed a sharp reduction of ETNPPL RNA in high-grade tumours compared to low-grade tumours (Fig. S4D)^14,19,20^. We confirmed this decrease by using IHC in a glioblastoma tumour. Result presented on Fig. 6C shows the absence of ETNPPL in this tumour. To validate this further, we used proteins extracted from one grade III astrocytoma and six GBM and compared with grade II tumours using WB. Figure 4A and Fig. S8A indicate that ETNPPL was absent or low in the 6 GBM samples whereas the protein was readily detected in 5 of the 7 non-GBM tumours.

Collectively, these data show that ETNPPL is expressed in diffuse low-grade tumours and is reduced with malignant progression.

### ETNPPL reduces glioma cell growth

**Figure 7:**
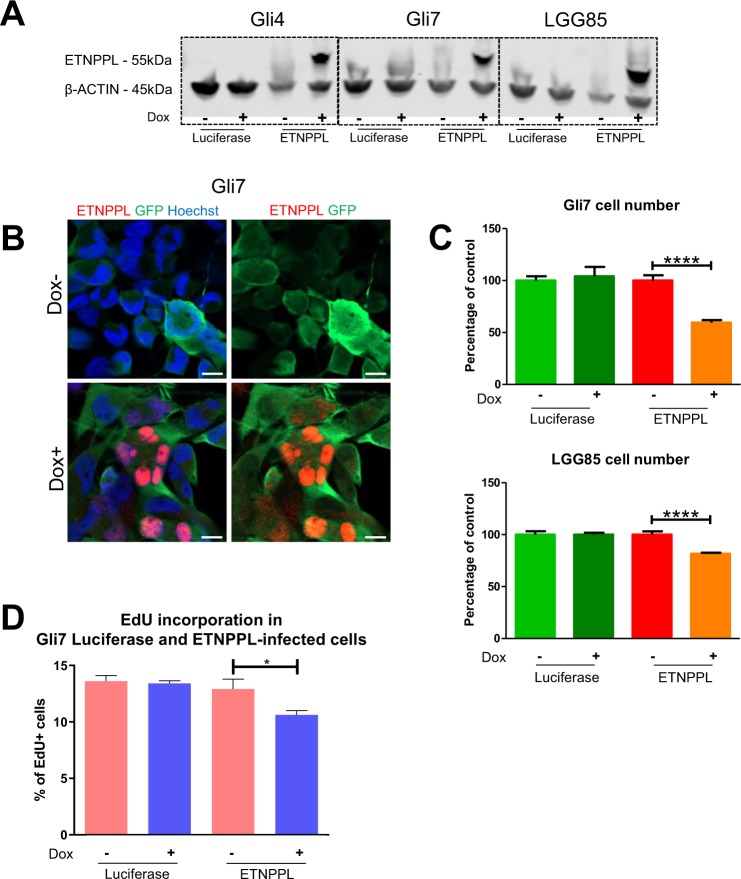
Functional assessment of ETNPPL expression in glioblastoma cell lines. (**A**) WB for ETNPPL in proteins extracted from Gli4, Gli7 and LGG85 cells infected with control (luciferase) or ETNPPL inducible-lentiviruses and cultured with and without doxycycline. The ETNPPL protein is only detected in ETNPPL-infected cells in the presence of doxycycline. β-actin detection is used as loading control. The uncropped image of the WB is presented on Supplemental Fig. 8B. **(B)** Immunofluorescence for ETNPPL in Gli7 cells infected with an ETNPPL lentivirus and cultured with and without doxycycline. ETNPPL (red) is strongly detected in the cell nuclei. Infected cells are detected by expression of the GFP (green) gene included in the lentiviral construct. **(C)** Representative experiment of the effect of ETNPPL overexpression on glioblastoma cell growth. Histograms show the relative number of Gli7 and LGG85 cells cultured for 8 days with and without doxycycline. Test = One-way ANOVA tests. n = 3 independent experiments. **(D)** Representative experiment of EdU incorporation in Gli7 cells. Histograms represent the % of EdU^+^ cells obtained after 4 hours of incorporation in the presence or absence of doxycycline. test = Mann-Whitney test (*p = 0.024), n = 3 independent experiments.

The reduction of ETNPPL in high-grade gliomas suggested that this enzyme might oppose proliferation which is enhanced in these tumours. We addressed this issue by performing gain of function experiments in three glioma cell cultures. We used two previously characterized glioma stem cell cultures (Gli4 and Gli7) which we derived from GBM-affected patients^11^. In order to use cells bearing mutations typically found in DLGG, we derived a third culture named LGG85 from a patient affected by a secondary GBM and containing a mutated IDH1 gene. LGG85 cells grow as neurospheres, express nestin (NES), an intermediate filament specific for neural immature cells, as well as two transcription factors highly expressed in gliomas (OLIG2, SOX2) (Fig. S5A,B). WB analysis and DNA sequencing show the expression of the IDH1 R132H protein in the cells and the IDH1 395 G > A mutation (Fig. S5C,D respectively). In addition, CGH array performed with the initial resection and with the LGG85 cells show a very good coincidence of DNA alterations between the tumour and the culture (Fig. S5E). ETNPPL was overexpressed in Gli4, Gli7, LGG85 cultures using a doxycycline-controlled lentivirus. Western blot analysis indicates that while endogenous ETNPPL in these 3 cultures was not detected (Fig. 7A), addition of doxycycline induced a strong ETNPPL expression compared to control luciferase-expressing cells. Immunofluorescence confirmed the induction of ETNPPL by doxycycline and the protein was localized in cell nuclei (Figs. 7B, S7B). Using these constructs in a growth assay, we found that ETNPPL overexpression reduced the cell number obtained after 8 days of culture in Gli7 and LGG85 cells (3 independent experiments, Fig. 7C), but not in Gli4 cells (not shown). EdU incorporation performed in Gli7 cells for 4 hours show a reduction in the proliferation rate (Fig. 7D) while the number of apoptotic cells detected by cleaved-caspase 3 was unchanged (not shown).

These results show an inhibitory role for ETNPPL in the growth of 2 out of 3 GBM cell lines.

## Discussion

In this article we characterized further the high-density foci observed in 20% of DLGG-affected patients by using IHC and RNA profiling. We provide evidence for dysregulation of specific pathways and gene expression in these tumour areas. In addition, this led us to identify expression of ETNPPL in gliomas, a barely-studied metabolic enzyme, which was further studied *in vitro*.

Three main conclusions can be drawn from this study.

### High activation of STAT3 pathway in foci

We first showed that foci have a higher percentage of pSTAT3^+^ cells which indicates STAT3 pathway activation in these cells. Phosphorylated STAT3 translocates to the cell nucleus to regulate many genes involved in proliferation, apoptosis and angiogenesis^21^. As such, phosphorylation of STAT proteins, notably STAT3, is involved in the pathogenesis of many cancers, including GBM, by promoting cell cycle progression, stimulating angiogenesis, and impairing tumour immune surveillance^21^.

What could activate the STAT3 pathway in foci? STAT3 is phosphorylated through activation of several receptors such as IL-6 cytokine receptor, c-met receptor, PDGFR and EGFR receptors. Indeed, EGFR is frequently co-expressed with phosphorylated STAT3 in high-grade gliomas^22^. We previously reported that EGFR staining was higher (Hirsch score) in foci than the rest of the tumour which may be linked to the STAT3 activation we observed in these tumour areas^9^. *In vitro* STAT3 can also be activated by cell confluence^23^ which may be somewhat related to its activation in foci where cell density is increased (Figs. 1A, S1).

What could be the significance of STAT3 activation in glioma foci? STAT3 expression promotes tumour incidence in combination with PDGF in a glioma mouse model^24^. Activated STAT3 also correlates positively with glioma grade^22,25^. STAT3 is essential for maintenance and proliferation of aggressive glioblastoma stem cells^26,27^ and is involved in the transition of glioblastomas toward the even more aggressive mesenchymal glioblastoma subtype^28^.

All together, these data support the notion that STAT3 activation in foci may be linked to malignant progression which warrants further investigations.

### Foci show dysregulated gene expression

A second outcome of this study is the identification of a modified expression of 13 genes which were downregulated in foci. Individual analysis of the literature for these genes reveals interesting features. ADCYAP1R1 (a receptor for PACAP) is a protein involved in cAMP production. High cAMP can oppose gliomagenesis^29^ so the observed decrease of *ADCYAP1R1* might promotes foci cell proliferation. Expression of 2 genes coding for metabolic enzymes (*ALDOC, ETNPPL*) was modified suggesting that foci might have an altered metabolism as often observed during cancer progression.

Gene analysis also suggests that foci cells are likely to have modifications in 2 pathways, namely Wnt and EGFR pathways. The Wnt signalling which is central in gliomagenesis^30^, may be affected in foci cells as we observed downregulation of 2 genes participating in the regulation of this pathway, namely *DAAM2*^31^ and *SFRP2*^32^. The reduction of *SFRP2* gene expression is particularly significant as this Wnt pathway inhibitor is often reduced by DNA hypermethylation in the progression of various cancers^33^. Regarding EGFR signaling, reduction of *MLC1*, a gene which favors EGFR degradation^34^, may leads to the higher EGFR expression we previously observed by IHC in foci^9^. The cytoskeleton might also be affected in foci as gene coding for ezrin (*EZR*) a linker protein between plasma membrane and actin cytoskeleton, is reduced. Finally, cell-cell communication is likely to be altered. This is illustrated by the decrease of *GJA1*, the gene coding for connexin 43, a major connexin protein of glial cells^35^.

The fact that the downregulated genes in foci are also decreased in high-grade tumours (Table S3) suggests that foci are cells undergoing transformation. In addition, for 3 of these genes, namely *MLC1*, *CST3* and *GJA1*, it has been demonstrated that they oppose tumorigenesis when overexpressed in glioma lines^34,36,37^ and thus they present tumour suppressor activity. Similarly, the reduction of expression of these 3 genes might promote foci expansion and transformation.

Further investigations are needed to evaluate whether other genes identified in this study behave in a similar way.

### ETNPPL negatively regulates glioma cell growth

The third series of data we report here concerns ETNPPL, a metabolic enzyme barely studied so far. We demonstrated that the ETNPPL protein is present in normal astrocytes as well as in diffuse low-grade glioma cells but is downregulated with malignant progression. The situation may be reminiscent of the liver context, where ETNPPL is expressed in normal tissue but is downregulated in hepatocarcinoma^38^. One unexpected finding was our observation of a nuclear localization for ETNPPL. This was observed in normal astrocytes, in glioma cells but also *in vitro* when the protein was overexpressed in glioblastoma stem cell cultures. In some glioma tumours, we also observed subpopulations of cells with ETNPPL in the cytoplasm. The nuclear or cytoplasmic expression of ETNPPL in gliomas can also be observed in the human protein atlas^39^ which validates our observation further (Fig. S4E). It is now established that metabolic enzymes can have non-metabolic roles and can act in the nuclei as transcriptional regulators (the so-called moonlighting phenomenon^40^). A similar situation may apply to ETNPPL which warrants further investigations.

We found that ETNPPL RNA and protein are reduced in foci cells and absent in glioblastomas. This is consistent with glioma database analyses showing that ETNPPL expression is inversely correlated to STAT3 and MKI67 (Fig. S4F) whose expression are higher in foci and glioblastomas. In addition, Kaplan-Meier analysis shows that patients with low expression of *ETNPPL* have lower overall survival (Fig. S4G). These observations suggested that this enzyme may oppose glioma cells proliferation. We demonstrated this hypothesis by overexpressing ETNPPL in 3 glioblastoma cell cultures (Gli4, Gli7, LGG85). Gli7 and LGG85 cells were sensitive to ETNPPL overexpression which reduced their growth while no effect was detected in Gli4 cells. These glioblastoma-derived cultures have different types of mutations. Notably, Gli4 cells have lost one copy of the *NF1* gene (unpublished data) which characterizes the mesenchymal subtype of glioblastomas^41^. This most aggressive subtype of glioblastoma may have a specific metabolism conferring insensitivity to ETNPPL.

How could ETNPPL reduce Gli7 and LGG85 cell growth? This enzyme catalyzes the breakdown of phosphoethanolamine, converting it to ammonia, inorganic phosphate and acetaldehyde (Fig. S4A). Phosphoethanolamine is a primary amine which has a critical role in the biosynthesis of membrane phospholipids, notably phosphatidylethanolamines which are enriched in the nervous tissue. Ethanolamine and its phosphorylated form phosphoethanolamine have been shown to be potent mitogens for cancer cell lines^42,43^. We can thus speculate that the high expression of ETNPPL found in diffuse low-grade gliomas and its overexpression done here in glioblastoma cells may reduce phosphoethanolamine concentration and phosphatidylethanolamine synthesis causing reduction in glioma cell growth. In support of this notion, magnetic resonance spectroscopy investigation found that IDH1-mutant tumours are characterized by decreased levels of phosphoethanolamine^44^.

Our study presents two limitations. First, we found only a limited number of dysregulated genes in foci. The suboptimal quality of RNA extracted from patient FFPE blocks may account for this restricted gene identification. Second, we cannot formally rule out that few of the genes which were identified are in fact not expressed by tumoral cells but by tumour environment cells such as microglia cells. However, this is unlikely as both quantifications of tumoral cells in the DLGG and its foci and bioinformatics analyses performed with the ESTIMATE approach did not reveal a different cellular composition in both compartments. In addition, literature review indicated that most of the genes are expressed by neural cells and, at least for ETNPPL, we have demonstrated that this gene is expressed by tumoral cells.

In conclusion, diffuse low-grade gliomas inexorably progress into high-grade tumours. Patients with foci have a reduced overall survival^9^ so it is important to characterize these areas with specific molecular tools. We found that the increase in pSTAT3 and the decrease in ETNPPL stainings characterize these foci which could help to detect cells progressing toward malignancy and thus to stratify patients.

## Supplementary information

Supplementary Dataset 1.
