# Supplementary material for: Transformation Foci in IDH1-mutated Gliomas Show STAT3 Phosphorylation and Downregulate the Metabolic Enzyme ETNPPL, a Negative Regulator of Glioma Growth
Source: Sci Rep. 2020 Mar 26;10:5504. doi: 10.1038/s41598-020-62145-1 (PMC7099072; doi:10.1038/s41598-020-62145-1)
Supplement: Supplementary file 1 — Supplementary Dataset 1. [file 41598_2020_62145_MOESM1_ESM.pdf]

Transformation Foci in IDH1-mutated Gliomas Show STAT3 Phosphorylation and Downregulate the Metabolic Enzyme ETNPPL, a Negative Regulator of Glioma Growth

Leventoux N[1,2], Augustus M[1], Azar S[1], Riquier S[3], Villemin JP[4], Guelfi S[1], Falha L[1], Bauchet L[1,5], Gozé C[1,6], Ritchie W[4], Commes T[3], Duffau H[1,5], Rigau V[1,7,9] and Hugnot JP[1,8,9]

SUPPLEMENTARY INFORMATION

Supplement figure legends

Supplemental figure 1. Foci in diffuse low-grade gliomas. (A) Representative photographs taken in the diffuse low-grade gliomas areas (DLGG) and in the foci of the eight tumors used for RNA profiling (four oligodendrogliomas and four astrocytomas). Sections were stained by hematoxylin and eosin. Scale bars=100 µm. **(B)** Quantification. Histograms represent the fold change (number of cells in foci / number of cells outside foci) observed in the eight tumors studied. Test= Mann-Whitney test (n=4 tumors).

Supplemental figure 2. Microdissection of foci. (A) Examples of IDH1 R132H IHC stainings with DAB (brown) in one DLGG and one foci area. Scale bars=100 µm. **(B)** Examples of areas removed for RNA extractions and profiling. Sections stained by hematoxylin and eosin. **(C)** Percentage of tumoral cells detected by IDH1 R132H IHC staining in the tumor (DLGG) and the foci in two patients (one grade II oligodendroglioma (OII) and one grade II astrocytoma (AII)). Five fields containing between 40 to 120 cells were counted in the tumour and its foci. Both areas contain a vast majority of tumoral cells and no significant differences can be detected between the DLGG and foci areas. **(D)** Evaluation of tumor and foci purity with ESTIMATE bioinformatics analysis. Results for the stroma and immune cells scores and tumor purity scores (between 0 and 1) for the 8 tumors and corresponding foci are presented. Ratio of foci/tumor purity scores show that the purity of both areas were very similar and no significant difference could be observed.

Supplemental figure 3. RNA profilings of diffuse low-grade glioma samples and foci. Unsupervised heatmap of hierarchical clustering based on 5,000 most-expressed genes in foci

and in the other part of the tumor (DLGG) in the 8 studied patients**.** This heatmap was generated with TAC 3.1.0.5 software.

Supplemental figure 4. Database analysis of ETNPPL. (A) Chemical reaction catalysed by the ETNPPL enzyme. **(B)** RNA for ETNPPL is mainly detected in the human liver and brain in the NCBI database (www.ncbi.nlm.nih.gov/gene/64850/). **(C)** ETNPPL is specifically expressed in astrocytes (Brain RNA seq database from Dr B Barres's lab, www.brainrnaseq.org - Zhang, *et al.* Purification and Characterization of Progenitor and Mature Human Astrocytes Reveals Transcriptional and Functional Differences with Mouse. *Neuron*. (2016). (**D**) ETNPPL RNA expression in different glioma grades from Rembrandt (www.betastasis.com/glioma/rembrandt/gene_expression_in_glioma_subtypes/), NCBI GDS1962 (https://www.ncbi.nlm.nih.gov/geoprofiles/?term=gds1962) and TCGA (https://omictools.com/web-tcga-tool) databases. Number of patients per tumor type and grade are indicated below each diagram. p-values are indicated on diagrams. Tests=Kruskal-Wallis with post hoc Dunn tests. **(E)** Examples of nuclear and cytoplasmic stainings for ETNPPL downloaded from https://www.proteinatlas.org/ENSG00000164089-ETNPPL/pathology/glioma#img (Patients id 3174 and 122 respectively) - Uhlén, *et al.* Tissue-based map of the human proteome. *Science*. (2015) - These images downloaded from the Human Protein Atlas are licensed under the Creative Commons Attribution-ShareAlike 3.0 International License. **(F)** An inverse correlation is observed between STAT3/MKI67 and ETNPPL RNA expression in gliomas (brain lower-grade glioma, TCGA Firehose legacy, RNA seq database, 283 patients). Pearson and Spearman correlation coefficients and p-values are indicated on the figures. The diagram utilized in this figure were provided by the cBioPortal (www.cbioportal.org) - Cerami *et al*. The cBio Cancer Genomics Portal: An Open Platform for Exploring Multidimensional Cancer Genomics Data. *Cancer Discovery*. (2012). **(G)** Kaplan-Meier survival curves of patients with a high level (fold change>1.5, in red) or a low level (fold change<-1.5, in blue) of ETNPPL mRNA. Patients with intermediate level of ETNPPL are in yellow. Groups of patients are indicated as well as p-values between groups. The diagram utilized in this figure was provided by the Georgetown Database of Cancer (G-DOC Plus, https://gdoc.georgetown.edu), a project of the Georgetown Lombardi Comprehensive Cancer Center designed to provide advanced translational research tools to the scientific community - Madhavan S *et al.* G-DOC: a systems medicine platform for personalized oncology, *Neoplasia.* (2011). Database used was REMBRANDT (n=541 patients, all grade of gliomas), https://www.ncbi.nlm.nih.gov/geo/query/acc.cgi?acc=GSE108476). mRNA quantification was performed using Affymetrix microarrays and the Kaplan-Meier curves were obtained with the probe 221008_s_at.

Supplemental figure 5. Characterization of the LGG85 culture. (A) Brightfield photograph of LGG85 cells grown as neurospheres. Scale bar =10 µm. **(B)** Immunofluorescences for the indicated antibodies in LGG85 cells. These cells express the mutated form of IDH1 (R132H) together with NESTIN, OLIG2 and SOX2. **(C)** WB for IDH1 R132H on proteins extracted from LGG85. A single band is present at the expected size. **(D)** Partial IDH1 gene sequence showing the C.395G>A mutation. **(E)** CGH array diagrams for the LGG85 cells and the tumor from which it was derived. Note the very good overlaps of DNA alterations observed in the cells and the initial tumor. Main LGG85 DNA alterations are gain of chromosomes 7, 8 and 9 (partial), partial loss of chromosome 10, 11, 13.

Supplemental figure 6. ETNPPL expression in tumoral cells. Examples of tumoral cells expressing the mutated form of IDH1 (IDH1 R132H, white arrowheads) and ETNPPL (yellow arrows) either in the nucleus **(A)** or the cytoplasm **(B)**. Red arrows show negative cells indicating staining specificity. These photographs were taken in one grade II oligodendroglioma. Scale bars 10 µm.

Supplemental figure 7. Analysis of ETNPPL expression in glioma cells with a second antibody (HPA072938) directed against another part of the protein. (A) WB for ETNPPL in proteins extracted from Gli7 infected with ETNPPL inducible-lentiviruses and cultured during 3 and 5 days with and without doxycycline and from Gli7 control cells. The ETNPPL protein is only detected in ETNPPL-infected cells in the presence of doxycycline. β-actin detection is used as loading control. The uncropped image of the WB is presented on supplemental figure 8C. **(B)** Immunofluorescence for ETNPPL in Gli7 control cells or Gli7 cells infected with an inducible-ETNPPL lentivirus and cultured with and without doxycycline. ETNPPL (green) is strongly detected in cell nuclei of doxycycline-treated cells. **(C)** Examples of tumoral cells expressing the mutated form of IDH1 (IDH1 R132H, white arrowheads) and ETNPPL (yellow arrows) either in the nucleus (right-hand image) or the cytoplasm (left-hand image). Red arrows show negative cells indicating staining specificity. These photographs were taken in one grade II oligodendroglioma. **(D)** Detection of ETNPPL (green) in one diffuse low-grade gliomas culture. The presence of tumoral cells is indicated by staining for the mutated IDH1 R132H protein (red). In this patient, the ETNPPL protein is detected in the cytoplasm (white arrows) or the nucleus (yellow arrows).

Supplemental figure 8. Uncropped WB for figures 4A ; 7A and supplemental figure 7A are presented. MW= molecular weights. **(A)** WB figure 4A. **(B)** WB figure 7A. **(C)** WB supplemental figure 7A.

Supplement table legends

Table S1: Patients description

Table S2: Antibodies and primers description

Table S3: Differential expression (grade III/II) from The Cancer Genome Atlas (TCGA) database of dysregulated genes identified in foci. Expression values for indicated genes in grade II-grade III oligodendrogliomas (OII-OIII, n= 108 and 76 cases respectively) and in grade II-grade III astrocytomas (AII-AIII, n= 87 and 98 cases respectively) were retrieved from the TCGA database. Fold changes (grade II/grade III) and p-values (grade III vs grade II) (Kruskal-Wallis with a Dunn post-hoc test) are indicated. n.s.= non significant.

Figure S1

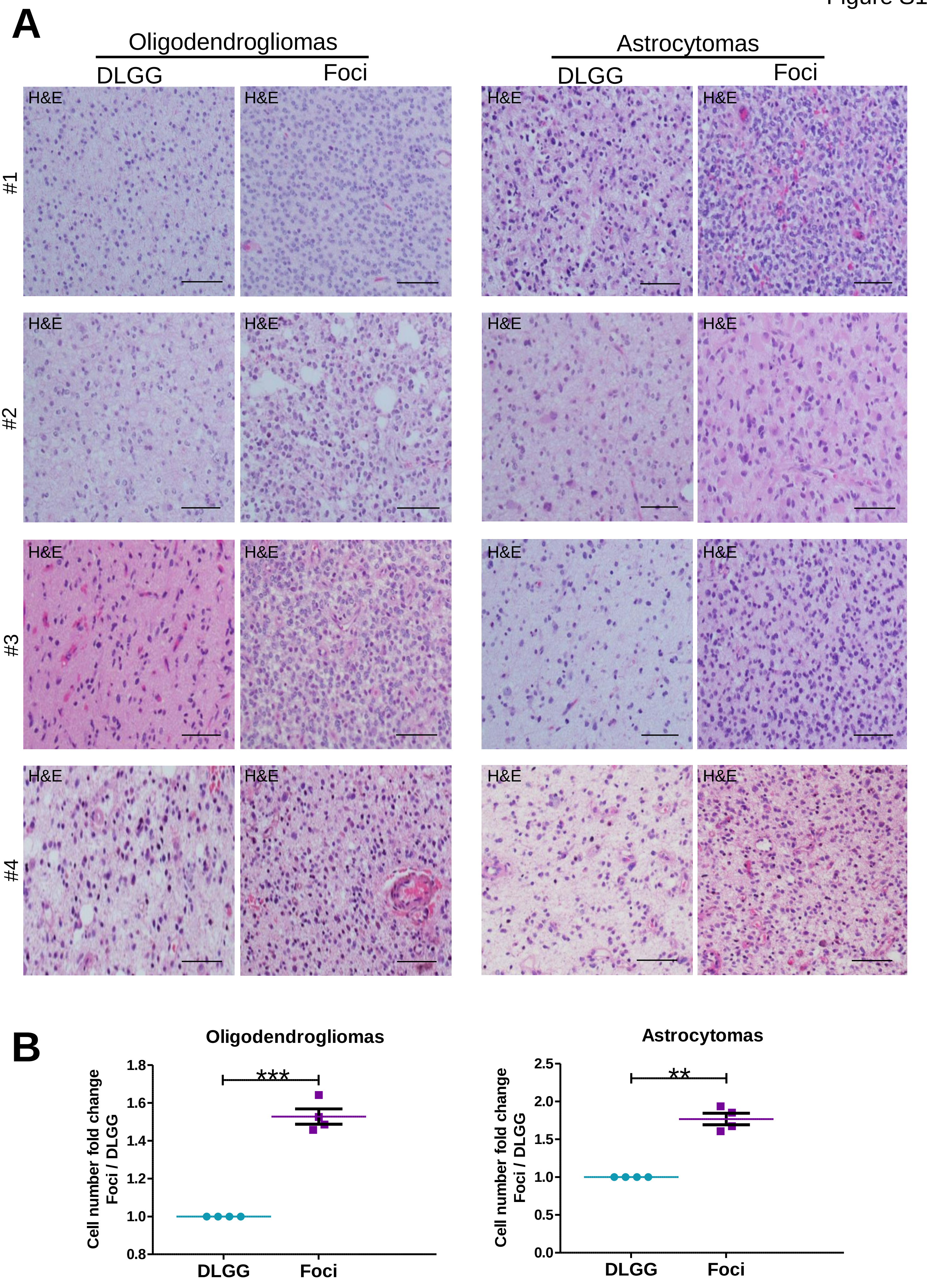
A
Oligodendrogliomas
DLGG
Foci
Astrocytomas
DLGG
Foci
H&E
#1
#2
#3
#4
B
Oligodendrogliomas
Cell number fold change Foci / DLGG
1.8
1.6
1.4
1.2
1.0
0.8
***
DLGG
Foci
Astrocytomas
Cell number fold change Foci / DLGG
2.5
2.0
1.5
1.0
0.5
0.0
**
DLGG
Foci

A

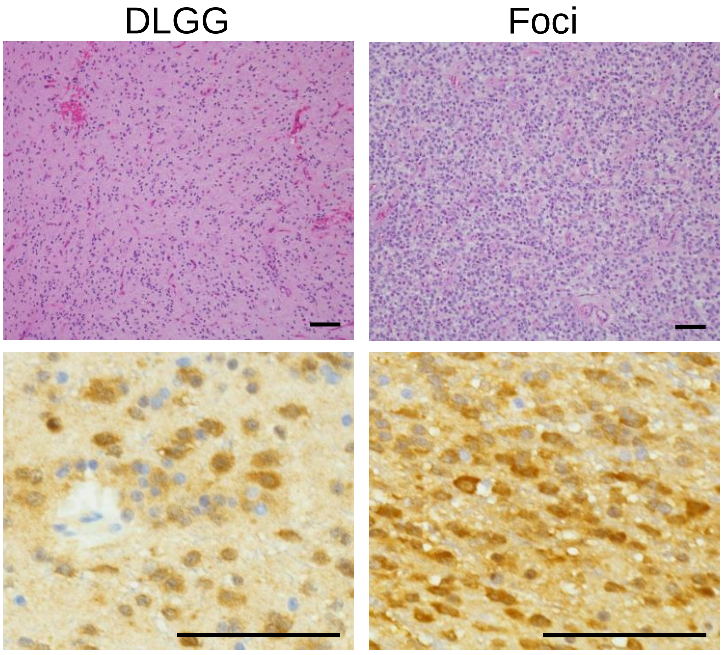

B

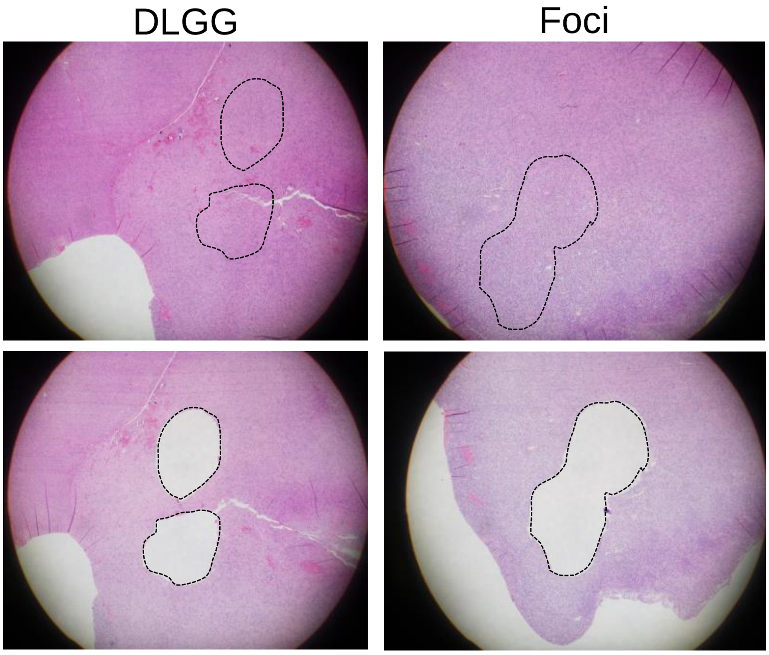

C

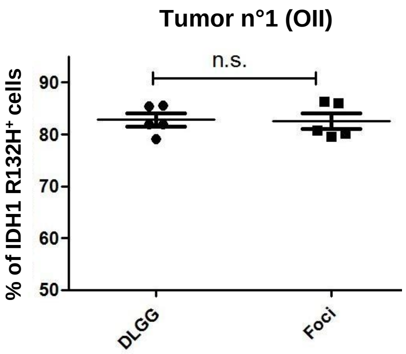

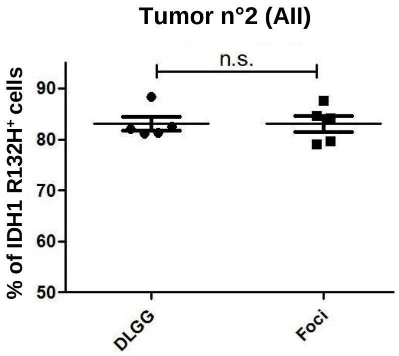

p=0.95, n=5 fields
Mann-Whitney test

D

SAMPLES	Tumor # 1	Foci # 1	Tumor # 2	Foci # 2	Tumor # 3	Foci # 3	Tumor # 4	Foci # 4	Tumor # 5	Foci # 5	Tumor # 6	Foci # 6	Tumor # 7	Foci # 7	Tumor # 8	Foci # 8
StromalScore	-739,6	-615,7	-313,7	-359,3	-869,9	-1 148,1	-375,0	-138,7	-867,4	-811,1	-793,6	-1 078,0	-986,9	-635,9	-391,1	-525,7
ImmuneScore	182,4	371,8	308,3	259,5	4,7	-336,0	256,8	442,0	-196,2	-65,1	-184,0	-399,8	-339,4	-42,7	266,5	205,7
ESTIMATEScore	-557,2	-243,9	-5,4	-99,8	-865,2	-1 484,0	-118,2	303,2	-1 063,6	-876,1	-977,7	-1 477,8	-1 326,3	-678,6	-124,6	-320,0
TumorPurity	0,87	0,84	0,82	0,83	0,89	0,93	0,83	0,80	0,90	0,89	0,90	0,93	0,92	0,87	0,83	0,85
TumorPurity ratios (foci/tumor)		0,97		1,01		1,04		0,96		0,99		1,03		0,95		1,02

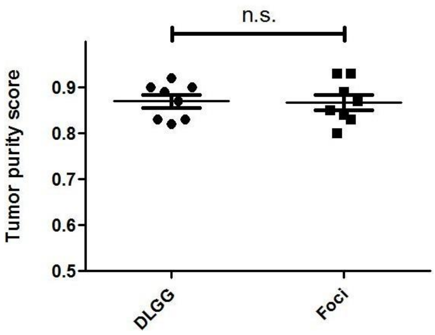

p=0.95, n=8 tumors
Mann-Whitney test

Figure S3

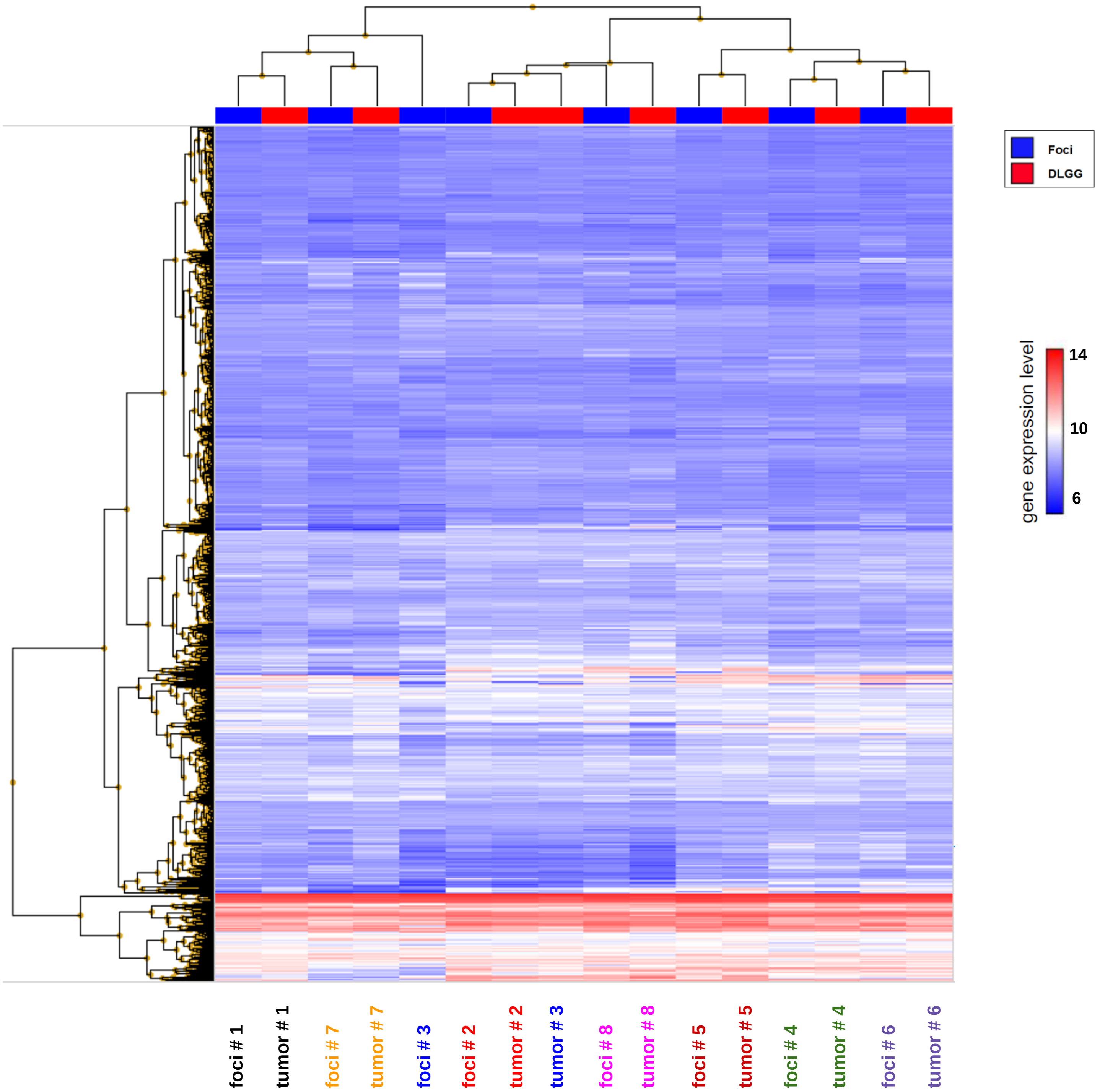

Foci
DLGG
14
10
6
gene expression level
foci # 1
tumor # 1
foci # 7
tumor # 7
foci # 3
foci # 2
tumor # 2
tumor # 3
foci # 8
tumor # 8
foci # 5
tumor # 5
foci # 4
tumor # 4
foci # 6
tumor # 6

Figure S4

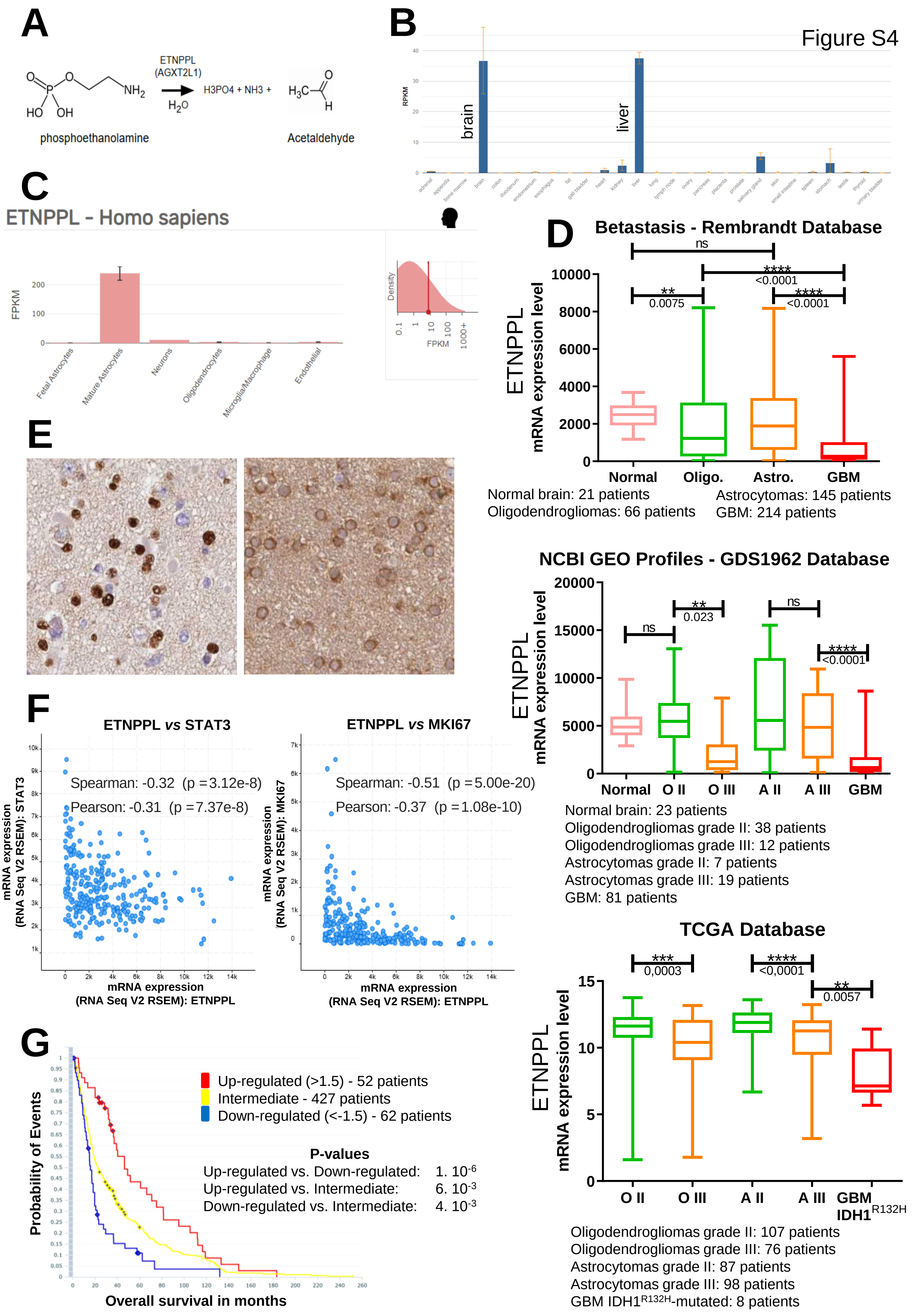
A
ETNPPL
(AGXT2L1)
H3PO4 + NH3 +
H2O
phosphoethanolamine
Acetaldehyde
B
RPKM
brain
liver
C
ETNPPL - Homo sapiens
FPKM
Fetal Astrocytes
Mature Astrocytes
Neurons
Oligodendrocytes
Microglia/Macrophage
Endothelial
Density
FPKM
D
Betastasis - Rembrandt Database
ETNPPL mRNA expression level
ns
****
<0.0001
**
0.0075
****
<0.0001
Normal
Oligo.
Astro.
GBM
Normal brain: 21 patients
Oligodendrogliomas: 66 patients
Astrocytomas: 145 patients
GBM: 214 patients
NCBI GEO Profiles - GDS1962 Database
ETNPPL mRNA expression level
ns
**
0.023
ns
****
<0.0001
Normal
O II
O III
A II
A III
GBM
Normal brain: 23 patients
Oligodendrogliomas grade II: 38 patients
Oligodendrogliomas grade III: 12 patients
Astrocytomas grade II: 7 patients
Astrocytomas grade III: 19 patients
GBM: 81 patients
TCGA Database
***
0,0003
****
<0,0001
**
0.0057
ETNPPL mRNA expression level
O II
O III
A II
A III
GBM IDH1R132H
Oligodendrogliomas grade II: 107 patients
Oligodendrogliomas grade III: 76 patients
Astrocytomas grade II: 87 patients
Astrocytomas grade III: 98 patients
GBM IDH1R132H-mutated: 8 patients
E
F
ETNPPL vs STAT3
Spearman: -0.32 (p =3.12e-8)
Pearson: -0.31 (p =7.37e-8)
mRNA expression (RNA Seq V2 RSEM): STAT3
mRNA expression (RNA Seq V2 RSEM): ETNPPL
ETNPPL vs MKI67
Spearman: -0.51 (p =5.00e-20)
Pearson: -0.37 (p =1.08e-10)
mRNA expression (RNA Seq V2 RSEM): MKI67
mRNA expression (RNA Seq V2 RSEM): ETNPPL
G
Up-regulated (>1.5) - 52 patients
Intermediate - 427 patients
Down-regulated (<-1.5) - 62 patients
P-values
Up-regulated vs. Down-regulated: 1. 10-6
Up-regulated vs. Intermediate: 6. 10-3
Down-regulated vs. Intermediate: 4. 10-3
Probability of Events
Overall survival in months

Figure S5

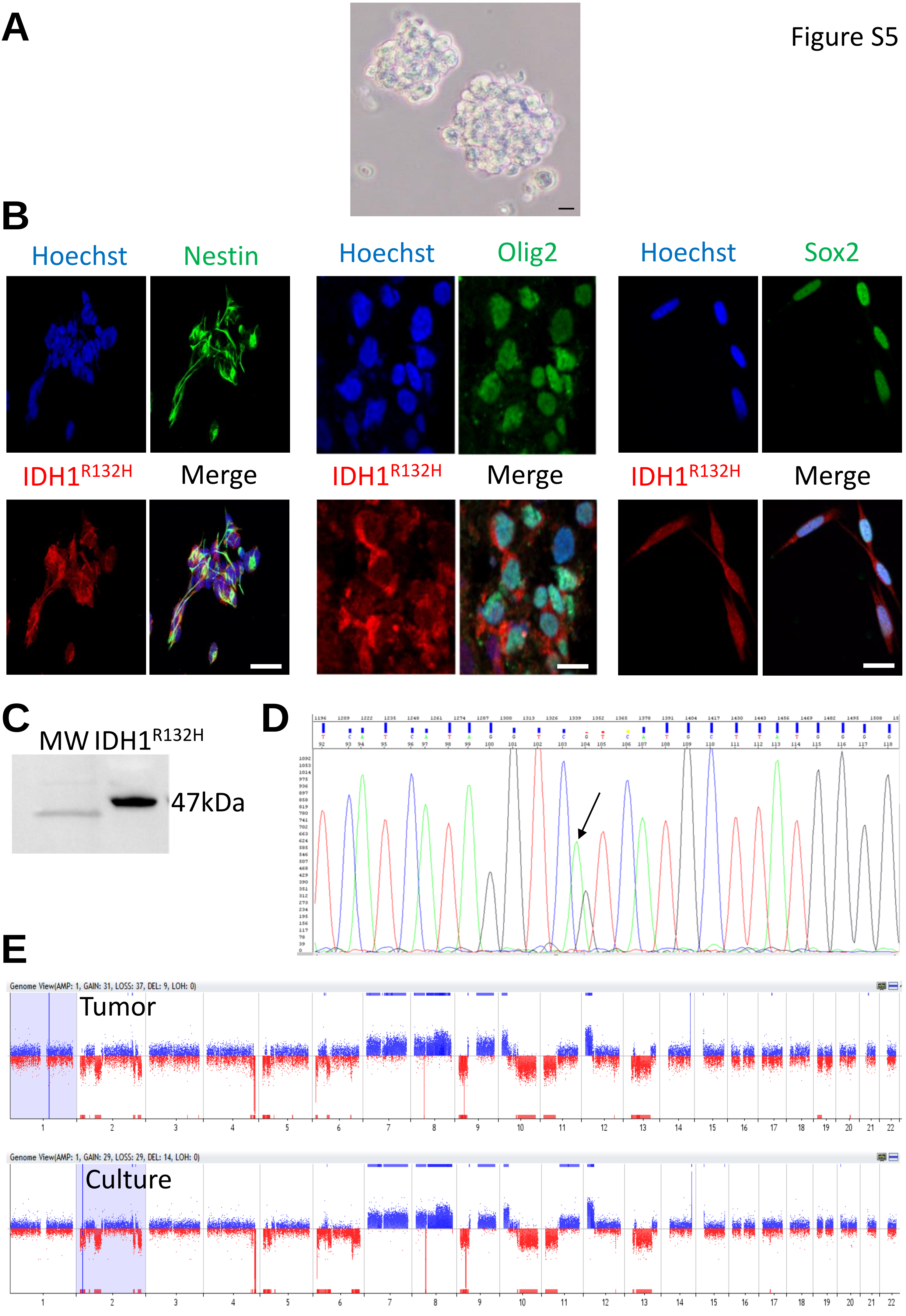
A
B
Hoechst
Nestin
Hoechst
Olig2
Hoechst
Sox2
IDH1R132H
Merge
IDH1R132H
Merge
IDH1R132H
Merge
C
MW IDH1R132H
47kDa
D
E
Genome View(AMP: 1, GAIN: 31, LOSS: 37, DEL: 9, LOH: 0)
Tumor
1 2 3 4 5 6 7 8 9 10 11 12 13 14 15 16 17 18 19 20 21 22
Genome View(AMP: 1, GAIN: 29, LOSS: 29, DEL: 14, LOH: 0)
Culture
1 2 3 4 5 6 7 8 9 10 11 12 13 14 15 16 17 18 19 20 21 22

Figure S6

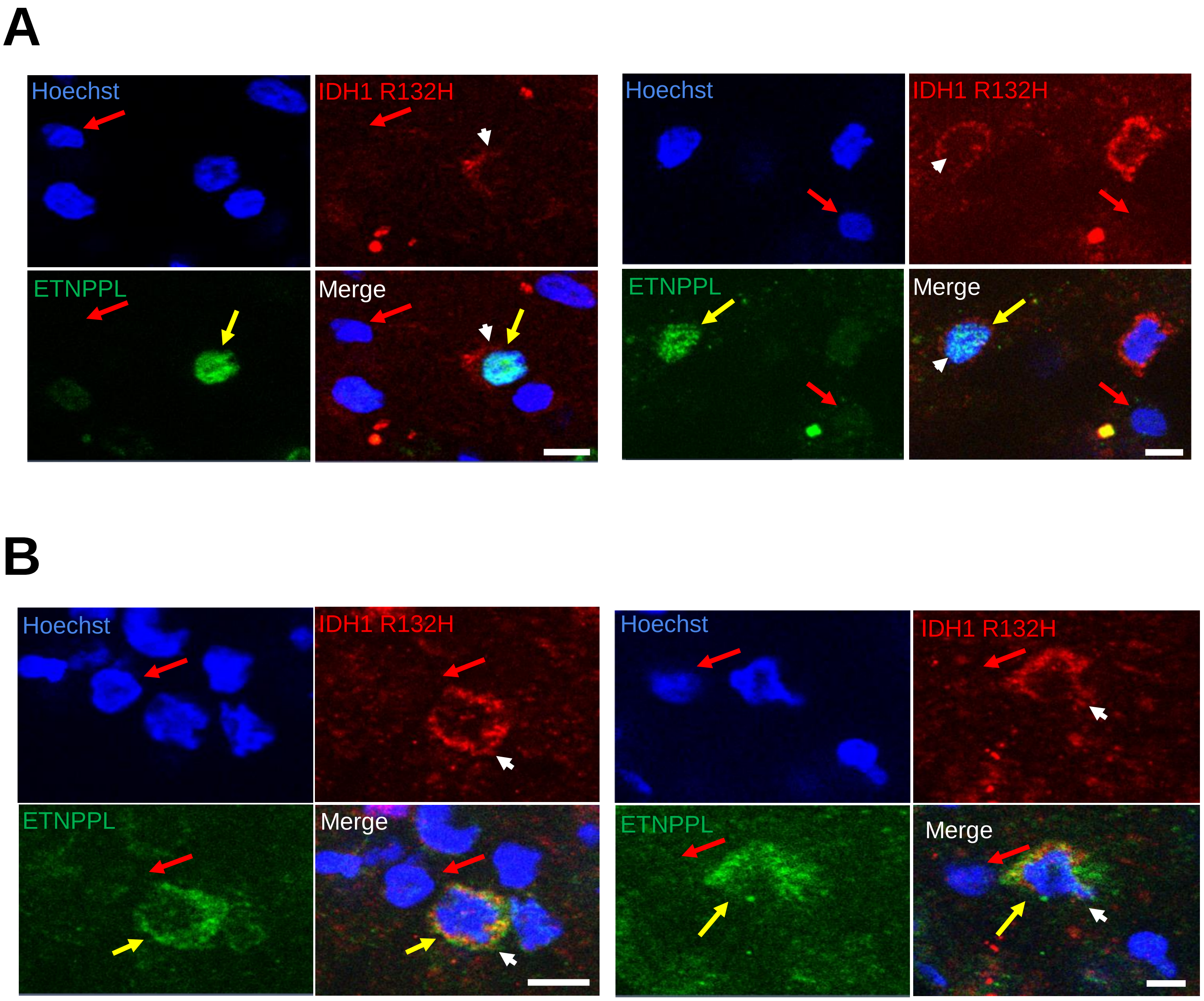
A
Hoechst
IDH1 R132H
ETNPPL
Merge
Hoechst
IDH1 R132H
ETNPPL
Merge
B
Hoechst
IDH1 R132H
ETNPPL
Merge
Hoechst
IDH1 R132H
ETNPPL
Merge

Figure S7

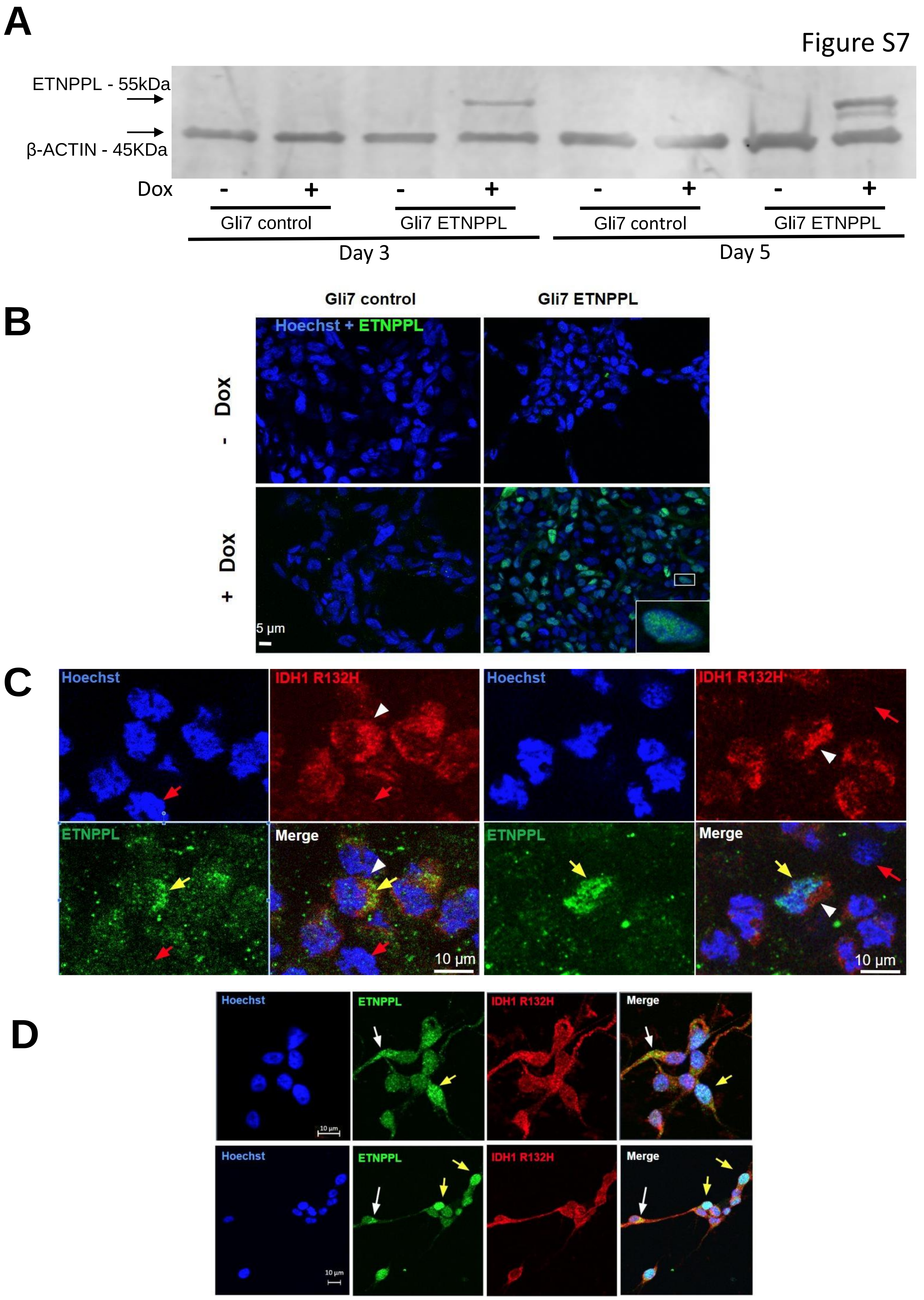
A
ETNPPL - 55kDa
β-ACTIN - 45KDa
Dox
- + - + - + - +
Gli7 control
Gli7 ETNPPL
Gli7 control
Gli7 ETNPPL
Day 3
Day 5
B
Gli7 control
Gli7 ETNPPL
Hoechst + ETNPPL
- Dox
+ Dox
5 µm
C
Hoechst
IDH1 R132H
Hoechst
IDH1 R132H
ETNPPL
Merge
ETNPPL
Merge
10 µm
10 µm
D
Hoechst
ETNPPL
IDH1 R132H
Merge
10 µm
Hoechst
ETNPPL
IDH1 R132H
Merge
10 µm

Figure S8

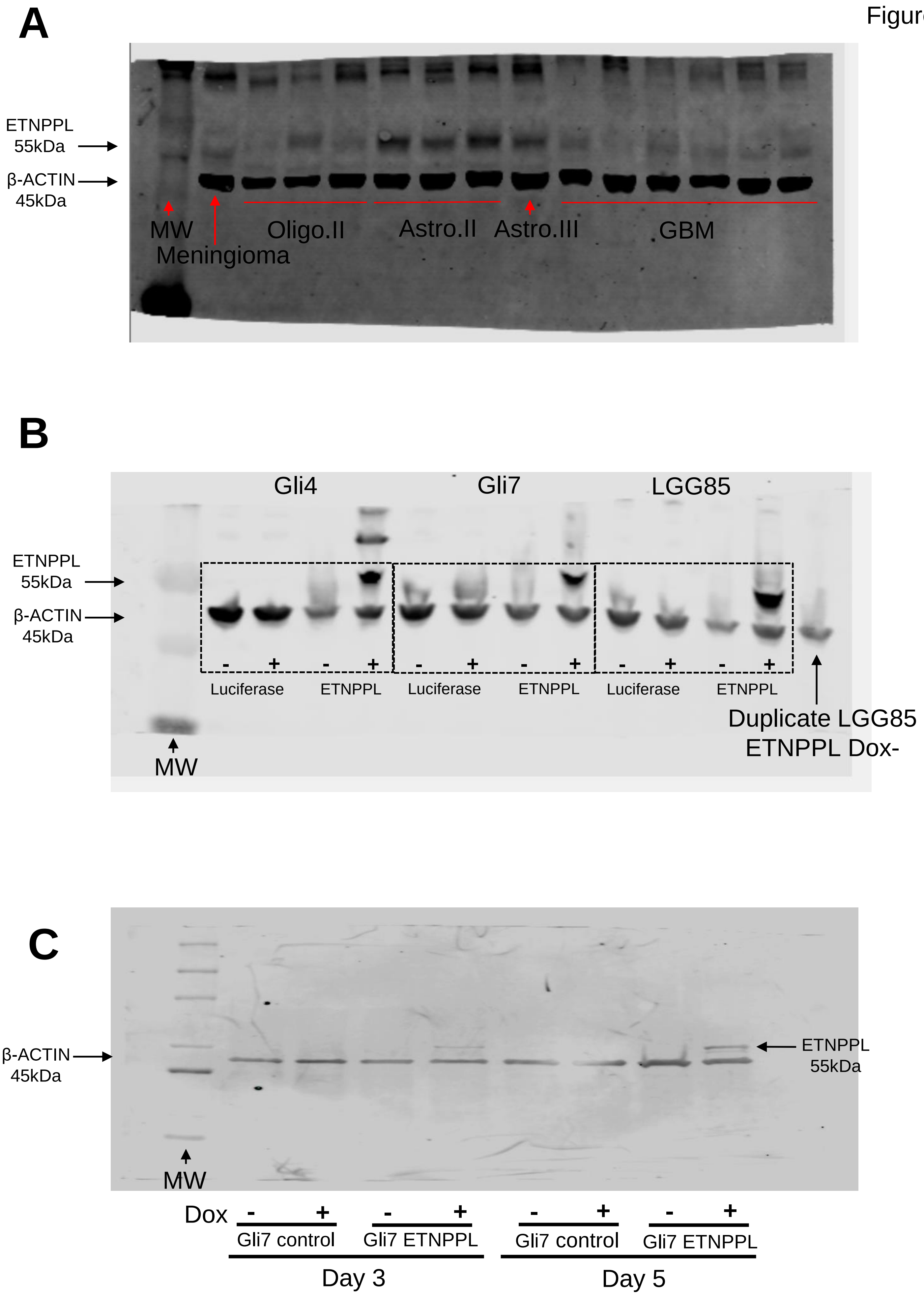
A
ETNPPL
55kDa
β-ACTIN
45kDa
MW
Meningioma
Oligo.II
Astro.II
Astro.III
GBM
B
Gli4
Gli7
LGG85
ETNPPL
55kDa
β-ACTIN
45kDa
- + - +
Luciferase
ETNPPL
Duplicate LGG85
ETNPPL Dox-
MW
C
β-ACTIN
45kDa
ETNPPL
55kDa
MW
Dox
- + - + - + - +
Gli7 control
Gli7 ETNPPL
Day 3
Day 5

Table S1

Patients description

(Histological and molecular features of the ten patients used for bioinformatics and foci studies)

Age at surgery	Sex	Histological subtype	1p19q status	IDH mutation	ATRX	Ki67 within foci
23 yo	F	Astrocytoma	1p no loss 19q partial loss	c.G395A Arg132His	loss	10%
21 yo	F	Astrocytoma	No loss	c.G395A Arg132His	loss	5%
27 yo	M	Astrocytoma	No loss	c.G395A Arg132His	loss	4%
23 yo	F	Astrocytoma	1p no loss 19q partial loss	c.G395A Arg132His	loss	15%
22 yo	M	Astrocytoma	No loss	c.G395A Arg132His	loss	8%
43 yo	M	Oligodendroglioma	Total loss	c.G395A Arg132His	maintained	20%
46 yo	M	Oligodendroglioma	Total loss	c.G395A Arg132His	maintained	20% focally
35 yo	M	Oligodendroglioma	Total loss	c.G395A Arg132His	maintained	3%
40 yo	M	Oligodendroglioma	Total loss	c.G395A Arg132His	maintained	10%
35 yo	M	Oligodendroglioma	Total loss	c.G395A Arg132His	maintained	15%

Patients description

(Histological and molecular features of the patients used for histological controls and DLGG comparison to foci)

Age at surgery	Sex	Histological subtype	1p19q status	IDH mutation	ATRX	Figure
38 yo	F	Glioblastoma	not available	not available	maintained	4B
43 yo	M	Oligodendroglioma	Total loss	c.G395A Arg132His	maintained	5A-C
35 yo	F	Astrocytoma	No loss	c.G395A Arg132His	loss	5B-C
32 yo	M	Astrocytoma	No loss	c.G395A Arg132His	loss	5B-C
27 yo	M	Astrocytoma	No loss	c.G395A Arg132His	loss	5D
43 yo	F	Astrocytoma	No loss	c.G395A Arg132His	loss	5D
41 yo	F	Oligodendroglioma	Total loss	c.G395A Arg132His	maintained	Sup 6A-B
44 yo	M	Oligodendroglioma	Total loss	c.G395A Arg132His	maintained	Sup 7C-D

Table S2

Antibodies description

Antibody	Manufacturer & Clone	Host Species	Dilution
Actine	Cell Signalling - 8H10D10	Mouse	1/6,000 for WB
ALDH1L1	Abcam ab56777	Mouse	1/50
ATRX	Santa Cruz - D5	Mouse	1/15
	Sigma HPA001906	Rabbit	1/500
CHI3L1	Santa Cruz - S18	Goat	1/50
ETNPPL	Sigma HPA044546	Rabbit	1/200 & 1/1,000 for WB
	Atlas Antibodies HPA072938	Rabbit	1/1,000 & 0.4µg/mL for WB
GFAP	Pharmingen - 1B4	Mouse	1/50
IDH1 R132H	Dianova - H09	Mouse	1/50
Vimentin	Dako - V9	Mouse	1/1,000
pSTAT3 - Tyr705	Cell Signalling - D3A7	Rabbit	1/200

Peptide	Manufacturer	Amino Acid Sequence	Dilution
ETNPPL	Novus - NBP1-91655PEP	VLKIKPPMCFTEEDAKFMVDQLDRILTVLEEAMGTKTE SVTSENTPCKTKMLKEAHIELLRDSTTDSKENPSRK	10 :1

Primers

Genes chosen to be assessed by RT-qPCR	Forward Primer	Reverse Primer	Amplicon size (bp)	Melting temperature
ALDOC	ACTCCATACCACAGCCCTTG	CAGCAATGAGAGAGGGGAAG	149	60°C
CST3	CCAGCAACGACATGTACCAC	CCTTTTCAGATGTGGCTGGT	170	60°C
ETNPPL	GCCGATGGACCTCATAGAAA	TTTCGGTTTTGGTTCCCATA	136	60°C
EZR	AGATCTGGGCAATGGATCTG	GCTGTCCTTTGCTGTGATGA	137	60°C
MLC1	TGCAGGACAGATCAGGTCAG	AACAGCGGAGATGGAGAAGA	132	60°C
SFRP2	CTTGGGTCTGGTTGGTTGTT	GGGCCACAGAGAAAATTGAA	152	60°C
SLC1A3	TAGTCCCGGTTTTGCATTTC	AATCTTGGCACACCAGAAGC	103	60°C
RPLP0	GCAATGTTGCCAGTGTCTGT	GCCTTGACCTTTTCAGCAAG	142	60°C

Table S3

Differential expression (grade III/II) from The Cancer Genome Atlas (TCGA) database of dysregulated genes identified in foci

Gene Symbol	Description	OII *vs* OIII		AII *vs* AIII	
		Fold Change	p-value	Fold Change	p-value
ADCYAP1R1	Adenylate Cyclase Activating Polypeptide 1 (Pituitary) Receptor Type I	1,68	0.0005	1,41	0.0363
ALDOC	Aldolase, Fructose-Bisphosphate C	1,40	0.0002	1,44	0.0031
ATP1A2	ATPase Na+/K+ Transporting Subunit Alpha 2	1,52	0.0012	1,69	0.0013
CST3	Cystatin C	1,12	ns	1,24	0.0477
DAAM2	Dishevelled Associated Activator Of Morphogenesis 2	2,00	< 0.0001	1,56	0.0008
ETNPPL	Ethanolamine-Phosphate Phospho-Lyase	2,40	0.0003	2,45	< 0.0001
EZR	Ezrin	1,41	0.0031	1,17	ns
GJA1	Gap Junction Protein Alpha 1	1,91	0.0010	1,67	0.0001
KCNN3	Potassium Calcium-Activated Channel Subfamily N Member 3	1,67	0.0039	1,25	ns
MLC1	Megalencephalic Leukoencephalopathy With Subcortical Cysts 1	1,73	< 0.0001	1,47	< 0.0001
SFRP2	Secreted Frizzled Related Protein 2	4,43	< 0.0001	2,95	< 0.0001
SLC1A3	Solute Carrier Family 1 Member 3	1,47	0.0002	1,32	0.0015
TMEM47	Transmembrane Protein 47	1,79	0.0001	1,52	< 0.0001
